# An Integrative Approach to Analyze Seed Germination in *Brassica napus*

**DOI:** 10.3389/fpls.2019.01342

**Published:** 2019-10-25

**Authors:** Marta Boter, Julián Calleja-Cabrera, Gerardo Carrera-Castaño, Geoffrey Wagner, Sarah Vanessa Hatzig, Rod J. Snowdon, Laurie Legoahec, Grégoire Bianchetti, Alain Bouchereau, Nathalie Nesi, Mónica Pernas, Luis Oñate-Sánchez

**Affiliations:** ^1^Centro de Biotecnología y Genómica de Plantas, (Universidad Politécnica de Madrid –Instituto Nacional de Investigación y Tecnología Agraria y Alimentaria), Madrid, Spain; ^2^Department of Plant Breeding, Justus Liebig University Giessen, Giessen, Germany; ^3^Joint Laboratory for Genetics, Institute for Genetics, Environment and Plant Protection (IGEPP), Le Rheu, France

**Keywords:** germination, seed traits, oilseed rape (*Brassica napus*), metabolism, hormonal pathways, transcriptomic, protein interaction, crop yield

## Abstract

Seed germination is a complex trait determined by the interaction of hormonal, metabolic, genetic, and environmental components. Variability of this trait in crops has a big impact on seedling establishment and yield in the field. Classical studies of this trait in crops have focused mainly on the analyses of one level of regulation in the cascade of events leading to seed germination. We have carried out an integrative and extensive approach to deepen our understanding of seed germination in *Brassica napus* by generating transcriptomic, metabolic, and hormonal data at different stages upon seed imbibition. Deep phenotyping of different seed germination-associated traits in six winter-type *B. napus* accessions has revealed that seed germination kinetics, in particular seed germination speed, are major contributors to the variability of this trait. Metabolic profiling of these accessions has allowed us to describe a common pattern of metabolic change and to identify the levels of malate and aspartate metabolites as putative metabolic markers to estimate germination performance. Additionally, analysis of seed content of different hormones suggests that hormonal balance between ABA, GA, and IAA at crucial time points during this process might underlie seed germination differences in these accessions. In this study, we have also defined the major transcriptome changes accompanying the germination process in *B. napus*. Furthermore, we have observed that earlier activation of key germination regulatory genes seems to generate the differences in germination speed observed between accessions in *B. napus*. Finally, we have found that protein–protein interactions between some of these key regulator are conserved in B. napus, suggesting a shared regulatory network with other plant species. Altogether, our results provide a comprehensive and detailed picture of seed germination dynamics in oilseed rape. This new framework will be extremely valuable not only to evaluate germination performance of *B. napus* accessions but also to identify key targets for crop improvement in this important process.

## Introduction

A plant´s life cycle starts with seed germination, a process aiming to produce a seedling able to grow, develop, and generate viable offspring. Germination is a crucial step for the establishment of crops in the soil and the uniformity of seedling emergence that, in turn, influence crop yield and quality. Seed germination is a complex trait influenced by different intrinsic (physiological and hormonal state of the seed) and external factors (environmental conditions during seed development, germination, and early seedling growth) as well as harvest and posterior storage (Basnet et al., 2015; Finch-Savage and Bassel, 2016). Thus, continuous interaction between environment and genotype allows seeds to germinate when conditions are suitable for plant growth (Kendall et al., 2011; Penfield and MacGregor, 2017). In crops, this trait seems to be controlled by several genes adding complexity to its study and their application in biotechnological and breeding programs. Moreover, seed germination encompasses a wide number of metabolic, hormonal, and molecular events aimed to timely produce the emergence of the embryo radicle through the surrounding seed tissues (Bewley, 1997; Holdsworth et al., 2008a). Therefore, improving germination in crops will require integrative approaches, combining a wide variety of global analyses (metabolic, molecular, and genomic analyses) with an array of computational methods.

Oilseed rape (OSR) is one of the world’s most important sources of high-quality vegetable oils for human nutrition and vegetable protein diets for livestock (Paterson et al., 2001). *Brassica napus* productivity is being challenged by variable environmental conditions that affect the reliability of seed germination in the field. Germination is a crucial trait in OSR production because seedlings must quickly attain self-sufficiency to survive. Previous studies in *B. napus*, focused mainly on the phenotypic and genetic aspects of germination traits, demonstrated that *B. napus* seed germination has substantial potential for improvement (Hatzig et al., 2015a). In this context, important efforts have been made to characterize the basic mechanisms regulating this trait using omic approaches, such as proteomics (Gu et al., 2016), hormonal profiling (Nguyen et al., 2016), oil content profiling (Ding et al., 2019), transcriptomics (Luo et al., 2019), and combination of microarray and proteomic data (Kubala et al., 2015). All these studies have been very useful to uncover some new regulatory mechanisms as well as to identify common mechanisms with other plant species. Nevertheless, an integration of all these data to provide a reliable framework to study and improve germination in this crop is still missing.

*B. napus* is closely related to the model crucifer *Arabidopsis thaliana*, and more than 86% of the protein coding sequences are conserved between them (Cavell et al., 1998). Most studies have taken advantage of the genetic and physiological proximity of both species to provide valuable guidance to analyze seed germination response in *B. napus* (Zhang et al., 2004). Nevertheless, there are clear differences between both species regarding seed size, seed composition, and seed dormancy. Moreover, *Arabidopsis* has not been subjected to the selection pressures of crop domestication, and therefore its seeds have not been challenged to perform in an agricultural context (Li et al., 2005; Finch-Savage and Bassel, 2016). Thus, it is expected that the mechanisms underlying the control of germination in this crop could be more complex and/or different to those found in *Arabidopsis*.

Seed germination begins with water uptake by the quiescent seed (imbibition) and ends with the elongation of the embryonic axis inside the seed. Seed imbibition triggers the reactivation of various metabolic, physiological, and biochemical processes necessary for the seed-to-seedling transition, such as resumption of respiratory activity, acquisition of energy, activation of repair mechanisms, protein biosynthesis from stored and newly synthesized mRNA, and reserve mobilization. Accordingly, initial catabolism of stored reserves of protein, oil, or starch accumulated during seed maturation will support the changes related with cell expansion, chloroplast development, and root growth that have to take place during this transition from heterotrophic to autotrophic nutrition (Holdsworth et al., 2008b; Rajjou et al., 2012). Many of these events are triggered and controlled by integration of environmental signals by hormones, mainly abscisic acid (ABA) and gibberellins (GAs), with antagonistic effects on the germination process (Shu et al., 2016). Environmental signals that are favorable or unfavorable for seed germination are associated with high GA/ABA or ABA/GA ratios, respectively. There is also a clear crosstalk between ABA and GAs since mutants with decreased or increased ABA content have altered expression of GA biosynthetic genes (Seo et al., 2006). An increased number of regulatory loci, additional hormones, and metabolic pathways have been identified as important for the germination process, and some of these components have been shown to work similarly in several plant species (Holdsworth et al., 2008a; Linkies et al., 2009; Morris et al., 2011; Rajjou et al., 2012; Shu et al., 2016). For instance, the role of ABA in regulating dormancy and germination through ABI3 and ABI5 (Graeber et al., 2010) and the function of GAs, PIL5, and DELLA proteins (Bassel et al., 2008; Davière and Achard, 2016; Zhou et al., 2017) are well established. However, to our knowledge, other important regulators discovered more recently, such as HFR1, NF-YC9, or NAC25, have not been investigated in the context of seed germination in plant species other than *Arabidopsis* (Shi et al., 2013; Liu et al., 2016; Ravindran et al., 2017; Sánchez-Montesino et al., 2019). Although all of these mechanisms would presumably be conserved in *B. napus*, differences in some of the regulatory aspects are expected to be different in the polyploid context of the crop genome. Other levels of regulation have also been shown to control germination in plants. Several studies have described global changes in mRNA populations during seed germination (Nakabayashi et al., 2005; Narsai et al., 2011; Narsai et al., 2017; Luo et al., 2019), adding the interaction of complex gene regulatory networks to the underlying mechanisms regulating this process. All these events have to be coordinated, temporally regulated, and integrated with environmental signals to accomplish a successful seedling establishment. Thus, combined metabolomic and transcriptomic approaches to integrate global transcriptional networks with metabolic and hormonal interactions are required to decipher this complex coordination.

For that reason, we have decided to build a framework to study seed germination traits in *B. napus*. We have used an integrative approach encompassing all of these different analyses based on data from six winter-type accessions (Hatzig et al., 2015). Accessions selected by their distinct germination rates were used to quantify hormones, metabolites, and RNA species at different stages upon seed imbibition. The robustness of the traits was confirmed by using seeds from glasshouse-grown plants to perform similar germination and seed trait assays. *In silico* analyses of all of these data sets together with quantitative expression analysis and protein–protein interaction assays have allowed us the identification of gene regulatory and hormonal networks as well as key genes that may explain the differences observed in germination traits between *B. napus* accessions. The germination framework reported here would be useful to define and evaluate targets that may be suitable for improvement of germination in OSR crops.

## Materials and Methods

### Plant Material and Growth Conditions

**Figure 1:**
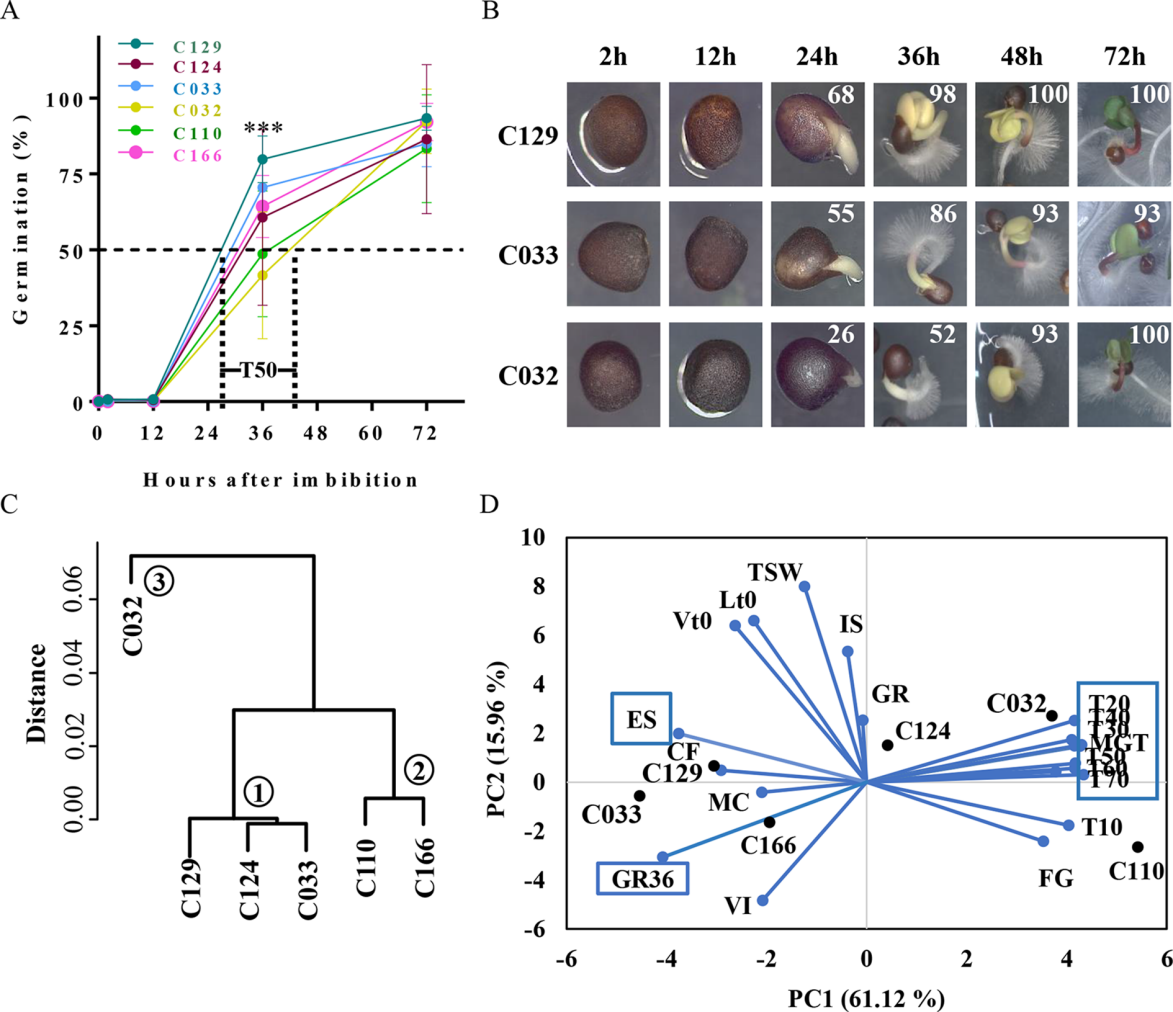
*B. napus* winter accessions show variability on seed germination kinetics. **(A)** WOSR accessions show significant differences in germination speed. Although most accessions started to germinate, defined as the radicle breaking through the testa, at 12 hai, main differences arise at T50 (required time to reach 50% of germination) with values that differ from 27 to 42 hai. The graph represents germination rates obtained for WOSR accessions C166, C129, C124, C033, and C032 for each time point tested (2, 12, 36, and 72 hai). **(B)** WOS accessions seeds with representative germination rates (high, medium, or low; C129, C033, and C032, respectively) were photographed during the time course to show visual differences in germination stages: seed imbibition (12 h), radicle breaking through the testa (24 h), radicle growth and cotyledon expansion (36 h), emerged seedling (48 h), and green seedling (72 h). Mean germination rates for each time point are written at the top right corner of each picture. **(C)** Dendrogram plot of WOSR accessions groups them in three clusters according to their germination kinetics. We used hierarchical clustering where Y-axes represent similarity based on Pearson correlation coefficients. **(D)** Biplot of principal component analysis (PCA) based on seed and germination traits shows that major contributors to variability in WOSR accessions (labeled in bold black) are seed germination kinetic parameters (blue boxes), whereas seed and seed imbibition traits show less contribution. Values were obtained from the GEVES automated phenotyping platform and measured for the C166, C129, C124, C110, C033, and C032 WOSR accessions. All traits are labeled in blue. All data were obtained using four biological replicates (25 seeds per replicate). For complete value data set, see **Supplementary Table S1**. GR, germination rate at 72 h; GR36, germination rate at 36 h; MGT, mean germination time; T10 to T70, time to reach 10% to 70% GR, respectively; FG, first germination time; ES, root elongation speed; MC, moisture content; IS, imbibition speed; VI, volume increase; CF, chlorophyll fluorescence; TSW, 10,000 seed weight; Lt0, seed length at time 0; Vt0, seed volume at time 0.

A total of six *B. napus* winter OSR five oil types and one fodder type) varieties showing contrasting vigor performance in previous experiments were used: C129, C032, C033, C166, C124, and C110 (Hatzig et al., 2015a). *B. napus* seeds were sown in MS (Murashige and Skoog) plates supplied with 1% sucrose. Plates were incubated in a germination chamber at 22ºC with 16-h light/8-h darkness photoperiod for 7 days before transferring seedlings to soil pots (3:1 peat-vermiculite fertilized with NPK 25-9-10 every 15 days). Soil pots were kept in a glasshouse with the same light and temperature regimes for 1 month, moved to a vernalization module (4–6ºC) for 2 months, and transferred to bigger soil pots (25 cm Ø) fertilized with NPKS 15-15-15-25 before moving them back to the glasshouse. Seeds were harvested from individual plants when siliques turned brown and stored in a dry and cold environment. For germination kinetics measurements (except **Figures 1A, B**), metabolic and hormone profile analyses, and RNAseq analyses, seeds were produced by controlled self-pollination in Asendorf, Germany, in field trials.

### Germination Assays and Seed Measurements

For germination kinetics measurements and metabolomic and hormone profile analyses, at least three biological replicates (50 or more seeds per replicate) were imbibed on moistened ﬁlter paper in Jacobsen germination vessels ﬁlled with 50 ml distilled water and germinated in a growth chamber at a constant temperature of 20°C and with a relative humidity between 50 and 75% in the dark. Material was collected, frozen, and ground up for further molecular analyses. For hormone profile analyses, the three biological replicates were pooled before measurements. Pictures were taken before material collection at time 0 (dry seeds), 2 h, 12 h, 36 h, and 72 h after imbibition to measure seed germination. Significant differences in germination performance among the varieties were confirmed by one-way and two-way ANOVA and Student’s t-test. Four biological replicates of 15 seeds (**Figure 1B**) and three biological replicates of 25 seeds of each accession were used for germination kinetics and qPCR analysis (**Figure 8**), respectively. All seeds were sown in 0.6% agarose plates and incubated in a germination chamber at 22ºC, with 16-h light/8-h darkness photoperiod. Germination was scored as radicle emergence through the endosperm and testa at different times after sowing. Significant differences in germination performance among the varieties were confirmed by one-way ANOVA and Student’s t-test.

Monitoring of seed imbibition, germination, and early radicle growth was conducted under *in vitro* conditions using the automated phenotyping platform of the variety control office of the French national seed testing agency (Station Nationale d’Essais de Semences, Groupe d’Etude et de contrôle des Variétés et des Semences—GEVES, Angers, France). The phenotyping platform is described in detail by Ducournau et al. (2004, 2005) and Wagner et al. (2011). Image acquisition, image analysis, and data analysis methods are described in detail by Demilly et al. (2014). The following parameters were determined: Volume increase within first 8 h (VI; in %), imbibition speed during first 4 h after initiation of imbibition (IS; in mm^3^/h), total germination rate within 72 h after initiation of imbibition (GR72; in %), first germination time (FG; in h), mean germination time (MGT; in h), radicle elongation speed (ES; in mm/h), time to reach 50% of germination (T50; in h), and germination rate within 36 h after initiation of imbibition (GR36; in %) (Hatzig et al., 2015a). Additionally, thousand seed weight (TSW; in g) was measured before germination monitoring. In total, 100 seeds per genotype were analyzed in four replicates (25 seeds per replicate). For seed length, seed volume, and seed area shown (**Supplemental Figures 1B, D**), three replicates of 100 seeds each from three different plants were measured. Seed images were processed using ImageJ (FIJI). Seed volume and area were inferred from the seed area, assuming seeds as spheres. Thousand seed weight (TSW) was determined by counting and weighting three replicates of 100 seeds each from three different plants.

### Metabolite Profiling

Metabolite analysis was performed on a Waters Acquity UltraPerformance Liquid Chromatography machine with diode array detection (Waters ACQUITY UPLC-DAD) using methods and software described in the Waters Corporation user manual. The manual was adapted for OSR tissue (Albert et al., 2012; Deleu et al., 2013). The AccQtag method was used to quantify amino acids, and the integration software Empower (Waters Corporation, Milford, USA) was used for analysis. Samples were resuspended in 100 ml distilled water. Subsequently, 5 ml were derivatized using AccQTag Ultra Derivatization Kit, according to the manufacturer’s recommendations. An external standard of 100 mmol/L of each amino acid was run every 10 samples. Quantification of sugars was performed using a Gas Chromatography-Flame Ionization Detector (GC-FID) System from Agilent Technologies (Santa Clara, CA, USA) according to (Lugan et al., 2009). The integrated Agilent software ChemStation Rev.B.04.02 was used for data analysis. Samples were resuspended in 50 ml pyridine (100%) with methoxamine hydrochloride (240 mmol/L), then derivatized with 50 ml MSTFA (N-methyl-N-(trimethylsilyl)trifluoro acetamide) (100%). An external standard containing 400 mmol/L of each sugar, sugar alcohol, and organic acid was run every 10 samples.

### Hormone Profiling

#### Chemicals and Calibration Curves

A number of compounds, namely, DPA, ABA-GE, PA, 7’- OH-ABA, neoPA, trans-ABA, and IAA-Glu were synthesized and prepared at the National Research Council of Canada, Saskatoon, SK, Canada; ABA, IAA-Leu, IAA-Ala, IAA-Asp, IAA, Z, ZR, iPR, and iP were purchased from Sigma–Aldrich; dhZ and dhZR were purchased from Olchemim Ltd. (Olomouc, Czech Republic); and GAs 1, 3, 4, 7, 8, 9, 19, 20, 24, 29, 44, and 53 were purchased from the Research School of Chemistry, Australian National University (Canberra, AU). Deuterated forms of the hormones that were used as internal standards include d3-DPA, d5-ABA-GE, d3-PA, d4-7’-OH-ABA, d3-neoPA, d4-ABA, d4-trans-ABA, d3-IAA-Leu, d3-IAA-Ala, d3-IAA-Asp, and d3-IAA-Glu were synthesized and prepared at NRCC SK according to Lulsdorf et al. (2013) and Zaharia et al. (2005); d5-IAA was purchased from Cambridge Isotope Laboratories (Andover, MA); d3-dhZ, d3-dhZR, d5-Z-O-Glu, d6-iPR, and d6-iP were purchased from Olchemim Ltd.; and d2-GAs 1, 3, 4, 7, 8, 9, 19, 20, 24, 29, 34, 44, 51, and 53 were purchased from the Research School of Chemistry, Australian National University. Calibration curves were created for all compounds of interest. Quality control samples (QCs) were run along with the tissue samples. Analysis was performed on a UPLC/ESI-MS/MS utilizing a Waters ACQUITY UPLC system, equipped with a binary solvent delivery manager and a sample manager coupled to a Waters Micromass Quattro Premier XE quadrupole tandem mass spectrometer *via* a Z-spray interface. MassLynx™ and QuanLynx™ (Micromass, Manchester, UK) were used for data acquisition and data analysis.

#### Extraction and Purification

An aliquot (100 µl) containing all the internal standards, each at a concentration of 0.2 ng µl^-1^, was added to homogenized sample (approximately 50 mg). Three milliliters of isopropanol:water:glacial acetic acid (80:19:1, v/v/v) were further added, and the samples were agitated in the dark for 14–16 h at 4ºC. Samples were then centrifuged, and the supernatant was isolated and dried on a Büchi Syncore Polyvap (Büchi, Switzerland). Furthermore, they were reconstituted in 100 µl acidified methanol, adjusted to 1 ml with acidified water, and then partitioned against 2 ml hexane. After 30 min, the aqueous layer was isolated and dried as above. Dry samples were reconstituted in 800 µl acidified methanol and adjusted to 1 ml with acidified water. The reconstituted samples were passed through equilibrated Sep-Pak C18 cartridges (Waters, Mississauga, ON, Canada), the final eluate was split in two equal portions. One portion (#1) was dried completely (and stored), whereas the other portion was dried down to the aqueous phase on a LABCONCO centrivap concentrator (Labconco Corporation, Kansas City, MO, USA). The second portion was partitioned against ethyl acetate (2 ml) and further purified using an Oasis WAX cartridge (Waters, Mississauga, ON, Canada). The GA-enriched fraction (#2) was eluted with 2 ml acetonitrile:water (80:20, v/v) and then dried on a centrivap as described above. An internal standard blank was prepared with 100 µl of the deuterated internal standards mixture. A quality control standard (QC) was prepared by adding 100 µl of a mixture containing all of the analytes of interest, each at a concentration of 0.2 ng µl^-1^, to 100 µl of the internal standard mix. Finally, fractions #1 and #2, blanks, and QCs were reconstituted in a solution of 40% methanol (v/v), containing 0.5% acetic acid and 0.1 ng µl^-1^ of each of the recovery standards.

### Hormone Quantification by HPLC-ESI-MS/MS

The procedure for quantification of ABA and ABA catabolites, cytokinin, auxin, and gibberellins in plant tissue was performed as described in detail in Lulsdorf et al. (2013). Samples were injected onto an ACQUITY UPLC® HSS C18 SB column (2.1 × 100 mm, 1.8 µm) with an in-line filter and separated by a gradient elution of water containing 0.02% formic acid against an increasing percentage of a mixture of acetonitrile and methanol (50:50, v/v).

Briefly, the analysis utilizes the Multiple Reaction Monitoring (MRM) function of the MassLynx v4.1 (Waters Inc.) control software. The resulting chromatographic traces are quantified off-line by the QuanLynx v4.1 software (Waters Inc.) wherein each trace is integrated and the resulting ratio of signals (nondeuterated/internal standard) is compared with a previously constructed calibration curve to yield the amount of analyte present (ng per sample). Calibration curves were generated from the MRM signals obtained from standard solutions based on the ratio of the chromatographic peak area for each analyte to that of the corresponding internal standard, as described by Ross et al. (2004). The QC samples, internal standard blanks, and solvent blanks were also prepared and analyzed along each batch of tissue samples.

### RNA Extraction, Library Construction, and Sequencing

The total RNA was extracted using the Nucleospin miRNA kit (Mecherey-Nagel, Düren, Germany). The protocol dedicated to the isolation of total RNA was used. RNA quality was assessed using the QIAxcel capillary electrophoresis (Qiagen, Hilden, Germany), and the concentration was measured using the Qubit 2.0 fluorometer (Life Technologies, Surrey, UK). Samples were stored at -80°C. For high-quality RNA extraction, three biological replicates (30 seeds each) were collected and pooled for each time point. cDNA libraries were constructed using the TruSeqnRNA Sample Prep Kit v2 (Illumina) and were sequenced on the Illumina HiSeq2000 platform at LGC Genomics (Berlin, Germany) with 100-bp paired-end sequencing. The TRUSEQ PE Cluster Kit v3 and the TruSeq SBS Kit v3 were used, respectively, for cluster generation and for sequencing. The RNAseq data reported in this article have been deposited in the public functional genomics data repository GEO (accession number GSE137230, https://www.ncbi.nlm.nih.gov/geo/query/acc.cgi?acc=GSE137230).

### Sequencing Data Analysis

Reads obtained from the sequencers were filtered to remove adapters. Reads were mapped to the reference genome (Genome assembly, AST_PRJEB5043_v1, Chalhoub et al., 2014) using HISAT. Assembly and quantification of transcripts in each sample were performed using StringTie. From all sets of probes obtained from each WOSR variety, we selected all probes that were seen in at least one time point (74,019 coding genes remained out of 101,040). Differentially expressed genes lists were obtained by pairwise comparison of all transcripts across samples and conditions [cutoff -1 > log_2_ fold change (FC) > 1]. For pipeline details, see Pertea et al., 2016.

### RNA Isolation and qRT-PCR

Ground tissue from 25 seeds was used for RNA isolation for each replicate (three biological replicates were used for each time point and accession). RNA extraction from seeds was carried out as described previously (protocol 2 in Oñate-Sánchez and Vicente-Carbajosa, 2008) with some modifications as described in Sánchez-Montesino et al. (2019). Total RNA samples were treated with DNase enzyme according to manufacturer’s protocol. Two micrograms of RNA were used for first-strand cDNA synthesis as described by Rueda-Romero et al. (2012) but using a mix of oligonucleotide dT (2.5 µM) and random hexamers (20 µM) as primers. Real-time qPCR conditions have been previously described (Rombolá-Caldentey et al., 2014) with the following modifications: i) annealing and extension temperature was 50°C; ii) expression values were normalized with those of 18S rRNA; iii) the 5x PyroTaq EvaGreen qPCR Mix Plus (Cultek Molecular Bioline) was used. Primers used for amplification of specific *B. napus* are listed in **Supplementary Table 3**.

### Protein–Protein Interaction Analyses

Specific full-length *B. napus* gene coding sequences (CDS) were amplified by two-step nested PCR as described in Castrillo et al. (2011). Primer sequences are listed in **Supplementary Table S3**. PCR products were subjected to electrophoresis, purified using the Geneclean Turbo Kit (MP Biomedicals) and cloned into a pDONR207 donor plasmid by a BP reaction (Invitrogen). CDSs in donor plasmids were transferred to the pGBKT7(GW) or pGADT7(GW) plasmids by an LR reaction (Invitrogen) to generate N-terminal translational fusions with the GAL4BD or GAL4AD CDSs, respectively, as specified in the text and figure legends. Integrity and quality of every construct were checked by sequencing. Constructs were introduced into the *Saccharomyces cerevisiae* pJ694a or YM4271A strains as described in Sánchez-Montesino and Oñate-Sánchez (2017, 2018). Alpha and A strains containing the BD or AD fusions, respectively, were used to interrogate for protein–protein interactions in a yeast two-hybrid system as described in Sánchez-Montesino and Oñate-Sánchez (2017, 2018).

### Statistics and Data Analysis

Hierarchical clustering was performed using the germination data shown in **Figure 1A** by the R software package with the HCLUST method using Pearson correlation distance as a metric. Correlation matrices were calculated for seed trait data from the automatic phenotyping platform and for hormone profile data (**Supplementary Table S1**, **Datasheet 1**, **3**) using R software and Pearson’s correlation coefficient as the statistical metric. To find positive and negative correlations between hormones and germination, the germination data shown in **Figure 1** was added as a continuous variable together with the hormone levels data. Principal component analysis (PCA) was performed using FactoMiner and factoextra R packages. Correspondence analysis shown in **Figure 6C** was performed using COA tool in MeV software (Chu et al., 2008). For k-means clustering of differentially expressed genes, we used Fuzzy K-means algorithm from XLSTAT software (Addinsoft) using a fuzziness coefficient of 1. Gene ontology analysis was performed using SeqEnrich software (Becker et al., 2017). Comparison of GOs obtained for each cluster was performed using SEACOMPARE tool included in AgriGO toolkit (Du et al., 2010).

## Results

### Seed Germination Speed Is a Key Contributor to Germination Variability in *B. napus* Winter Accessions

Rapid and uniform seed germination is key for seedling establishment and high crop yield. Variability in seed germination has been reported previously of *B. napus*, but a framework to analyze such differences has not yet been fully established. To define this framework, we performed a detailed analysis of the germination kinetics of six winter-type *B. napus* accessions (WOSR) selected by their variability in terms of germination. Germination in the selected WOSR accessions started as early as 12 h after imbibition (hai), although the first germination event (the radicle breaking through the testa) was observed predominantly at ∼24 hai (**Figures 1A, B**). In fact, we observed that most of the variability in the germination traits measured in these WOSR accessions arises at early time points. Thus, 50% of germination (T50) in these varieties was observed in a period spanning from 27 hai (C129) to 42 hai (C032) while final GRS (GR at 72 hai, end of germination for WOSR) varied from 93% (C129) to 83% (C110) (**Figure 1A**). In most of the WOSR accessions, cotyledons were visible at 36 hai, fully developed at 48 hai, and the greening of cotyledons was observed at 72 hai (**Figure 1B**). Although these results define a common developmental pathway during germination for all of the WOSR accessions analyzed, significant deviations from mean GRS were observed between 24 and 48 hai (**Figure 1A**). Specifically, at 36 hai, some accessions (C129, C124, and C033) were reaching 70% GR, whereas other lines (C032 and C110) were still close to just 40% GR (**Figure 1A**), suggesting a difference between accessions in germination kinetics rather than in their final GRS. To find key germination traits responsible for this variability, we first categorized the accessions based on their germination kinetics by hierarchical clustering. This analysis separated the accessions into three clusters (**Figure 1C**). C129, C124, and C033 grouped together (Cluster 1) and corresponded to accessions with faster germination speed (high GR at 36 hai in **Figure 1A**), whereas C110 and C166 with an intermediated germination speed grouped in a different category (Cluster 2). C032, showing the slowest germination speed (Low GR at 36 hai in **Figures 1A, B**), was the accession showing the greatest distance from clusters 1 and 2 and therefore constituted a separated group (Cluster 3). This clustering again supported the conclusion that the major germination trait responsible for WOSR variability is their germination speed. Then, we further characterize germination variability of these accessions using an automated phenotyping platform that allowed us to estimate a higher number of germination traits (**Supplementary Table S1**, **Datasheet 1**; Ducournau et al., 2004; Ducournau et al., 2005; Wagner et al., 2011; Demilly et al., 2014; Hatzig et al., 2015a). We use all of these measurements to assess the influence of each of these traits in the seed germination variability observed in the accessions by performing a PCA as well as a correlation analysis. As shown in **Figure 1D**, accessions with fast (C129 and C033) and slow (C032) germination are clearly separated on the horizontal axis, representing the first principal component (PC1). Furthermore, the PC1 explained a high percentage (61.12%) of the observed variability in the accessions, and the variables with major contribution to PC1 were all germination traits related to kinetics, GR36 (6.74%), T20 to T70 (50.4%, altogether), and mean germination time (MGT; 7.07%). Accordingly, high GR36 and low T20–T70 were observed for C129 and C033 while high T50 and MGT were measured for C032. Interestingly, PCA analysis highlighted root elongation speed (ES) as a trait with significant negative correlation with T50 (5.73% contribution to the PC1; **Figure 1D** and **Supplementary Figure S1A**). Consequently, accessions with higher germination speed (C129 and C033) showed higher ES. The second component (PC2) only explained 15.96% of the observed variability in the samples and was mainly related with seed associated traits like thousand seed weight (TSW), seed length at time 0 (Lt0), and seed volume at time 0 (Vt0), as well as with initial water uptake parameters like seed imbibition speed (IS), moisture content (MC), or seed volume increase (VI). Although these traits contributed to the overall phenotypic variation observed in the accessions used in this study, they were not significantly correlated with germination-associated traits like GR36 or T50 (**Supplementary Figure S1A**). Moreover, when we directly quantified these seed parameters in WOSR accessions with high, medium, or low germination rates (C129, C033, and C032, respectively), we did not observe clear correlation either. For instance, although C129 produces bigger seeds than C032 and C033, C129 and C032 showed similar TSW values. Moreover, C129, C033, and C032 have different values of seed volume, area, and length (C129 > C032 > C033) that also do not correlate with their GRS (**Supplementary Figures S1B–E**). Together, all of these results suggested that phenotypic variability related to germination in *B. napus* accessions relied primarily on the speed of germination rather than on final GRS.

Germination is a trait that needs to be addressed at different levels to get an insight of the major contributors to its performance. For this reason, we decided to make an integrative study comprising metabolic, hormonal, and transcriptional analyses to find the most relevant biological processes underlying the observed seed germination variability in *B. napus*. Based on hierarchical clustering and the previous multivariate germination analysis, we selected C129 and C033 as accessions showing a high-medium germination performance and C032 as an accession with low germination performance to carry out all of these analyses.

### Metabolomic Profile of Different WOSR Accessions During Germination Defines a Common Pattern of Metabolic Change in *B. napus*

*B. napus* seeds sustain intense desiccation by the end of their maturation period and retain their germination potential over long periods of dry storage (Roberts, 1973). To accomplish this, specific mechanisms maintain the state of metabolic quiescence in mature dry seeds to ensure that cell metabolism is activated and restarted during germination (Rajjou et al., 2012). Storage compounds are key for metabolism activation during seed imbibition and along the germination process to sustain early seedling growth (Weitbrecht et al., 2011; Nonogaki, 2014; Paszkiewicz et al., 2017).

**Figure 2:**
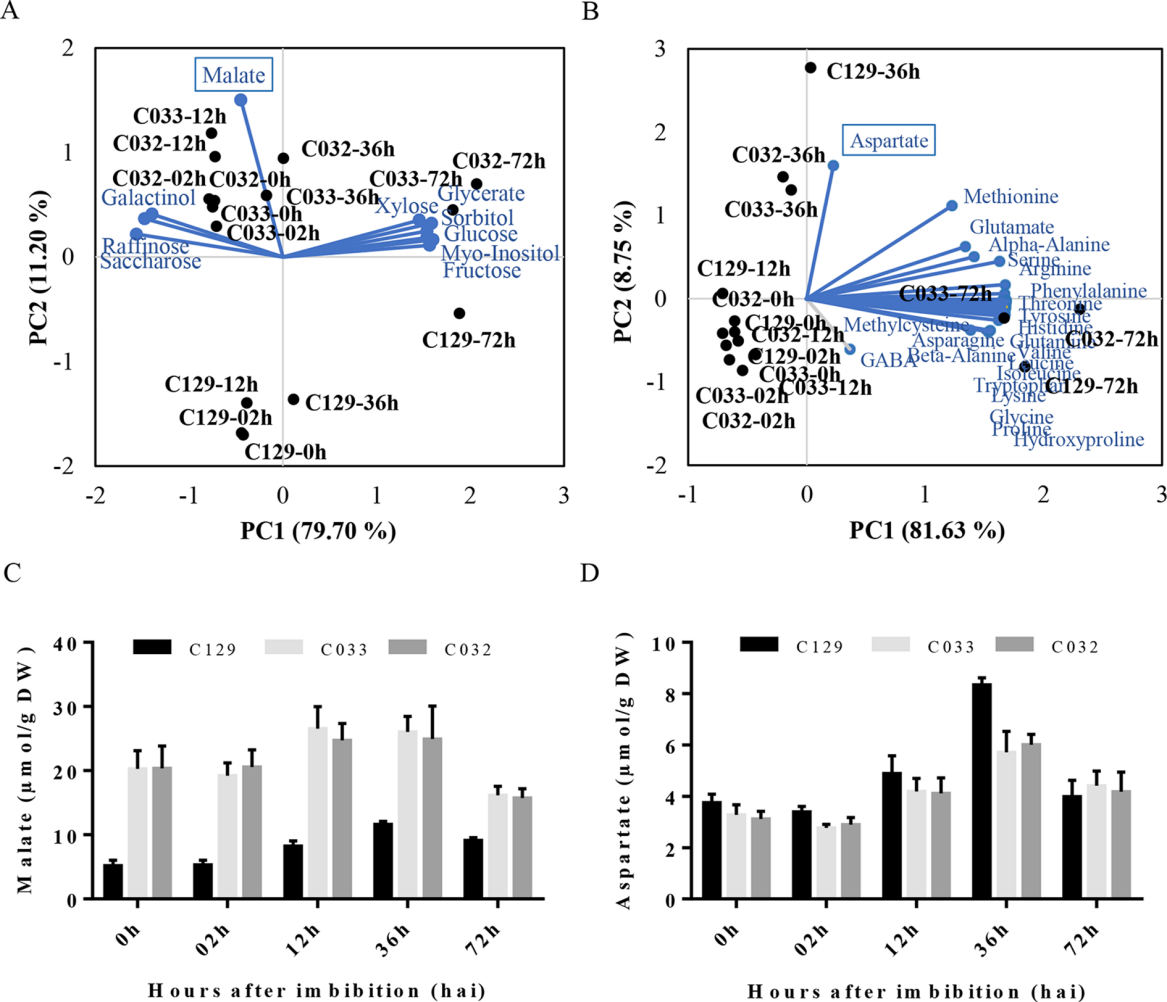
Metabolic profiles of winter oilseed rape (WOSR) accessions show that malate and aspartate levels correlate with germination performance. **(A)** Principal component analysis (PCA) of sugar, polyalcohol, and organic compound profiles at distinct developmental stages during seed germination reflects the requirement of metabolic energy during this process. Levels of malate (blue box) contribute to differential germination rates of C129 from 0 to 36 hai (blue dashed circle). WOSR accessions are labeled in black and sugar metabolites in blue. **(B)** Amino acid levels increase during germination in all accessions tested. Amino acid dynamics in WOSR accessions during germination represented as a biplot of PCA based on amino acid profiles and seed germination rates. Aspartate (blue box) has a differential accumulation, especially in C129 at 36 hai. WOSR accessions are shown in black and sugar metabolites in blue. **(C)** C129 has lower malate levels compared to C032 and C033. Quantification of malate (µmol/g DW) at 0, 12, 24, 48, and 72 hai in C129, C032, and C033 accessions is shown. **(D)** C129 has higher aspartate levels compared to C032 and C033. Quantification of aspartate levels (µmol/g DW) at 0, 12, 24, 48, and 72 hai in C129, C032, and C033 accessions is shown. All data were obtained using three biological replicates (50 seeds per replicate). For data of all metabolites, see **Supplementary Table S2**.

To get a global view on the metabolic changes that occur during seed germination, we analyzed the sugar, polyalcohols, organic compounds, and amino acid content in our WOSR accessions at key developmental times during seed germination (**Supplementary Table S1**, **Datasheet 2**). Then, we used PCA in our panel of selected accessions to identify the main contributors to the variability in germination related to these metabolites. First, we analyzed sugars and polyalcohols, and we identified sucrose, raffinose, and galactinol as main carbohydrate forms accumulated from 0 (dry seeds) to 12 hai. These results are consistent with previously published sugar models in *B. napus* where it was established that as the embryo develops, sucrose levels increase associated with degradation and mobilization of the reserves accumulated during seed maturation (Hill et al., 2003; Schwender et al., 2003). At 36 hai, we observed that those sugars started to decrease and monosaccharides (fructose, glucose, and xylose) and polyalcohols (myo-inositol and sorbitol) started to accumulate similarly to other seed germination profiles in other plants (Howell et al., 2008; Gu et al., 2016). Interestingly, PCA analysis of carbohydrates evidenced a clear separation of accession C129 based on malate content during germination. Detailed examination of malate levels revealed that the rapid-germination C129 has significantly lower levels of malate at all time points analyzed (**Figures 2A, C**). As malate is a key intermediate of the tricarboxylic acid (TCA) and glyoxylate cycle (Eastmond and Graham, 2001), we speculate that the better seed germination performance could be related to a more efficient mobilization of seed storage compounds to supply energy for seed germination and early seedling development. Second, we analyzed the amino acids levels in all accessions. We found a global and similar increase in all of the amino acids detected, accumulating during the progression of germination as described for seeds of other species (Fait et al., 2006) (**Figure 2B**). Interestingly, the PCA analysis highlighted a peak of aspartate at 36 hai. In this case, C129 reached higher levels of aspartate than C032 and C033 (**Figure 2D**), which correlates with higher levels observed for other accessions in cluster 2 (**Supplementary Table S1**, **Datasheet 2**). Aspartate is, with other amino acids, part of the metabolites required for the TCA cycle (Weitbrecht et al., 2011), suggesting that higher levels of aspartate could also be related to better energy efficiency during germination. In summary, results from our analysis on metabolic profiles of *B. napus* accessions agree with previous data obtained for other crops and point to malate and aspartate as putative metabolic markers to estimate germination performance.

### Comparative Hormonal Profiling Highlights Hormonal Balance as a Key Process Determining Germination Performance in *B. napus*

Germination has been found to be under strict regulation of plant hormones, mainly GAs and ABA, with the involvement of other hormones, such as auxin and cytokinins (Finkelstein et al., 2008; Han and Yang, 2015; Shu et al., 2016). Generally, ABA biosynthesis and sensitivity increase during seed development and maturation to prevent premature germination, whereas GA accumulation and sensitivity dictate germination after seed imbibition, promoting the transition to germination (Holdsworth et al., 2008a; Rajjou et al., 2012; Nonogaki, 2014; Vishal and Kumar, 2018).

**Figure 3:**
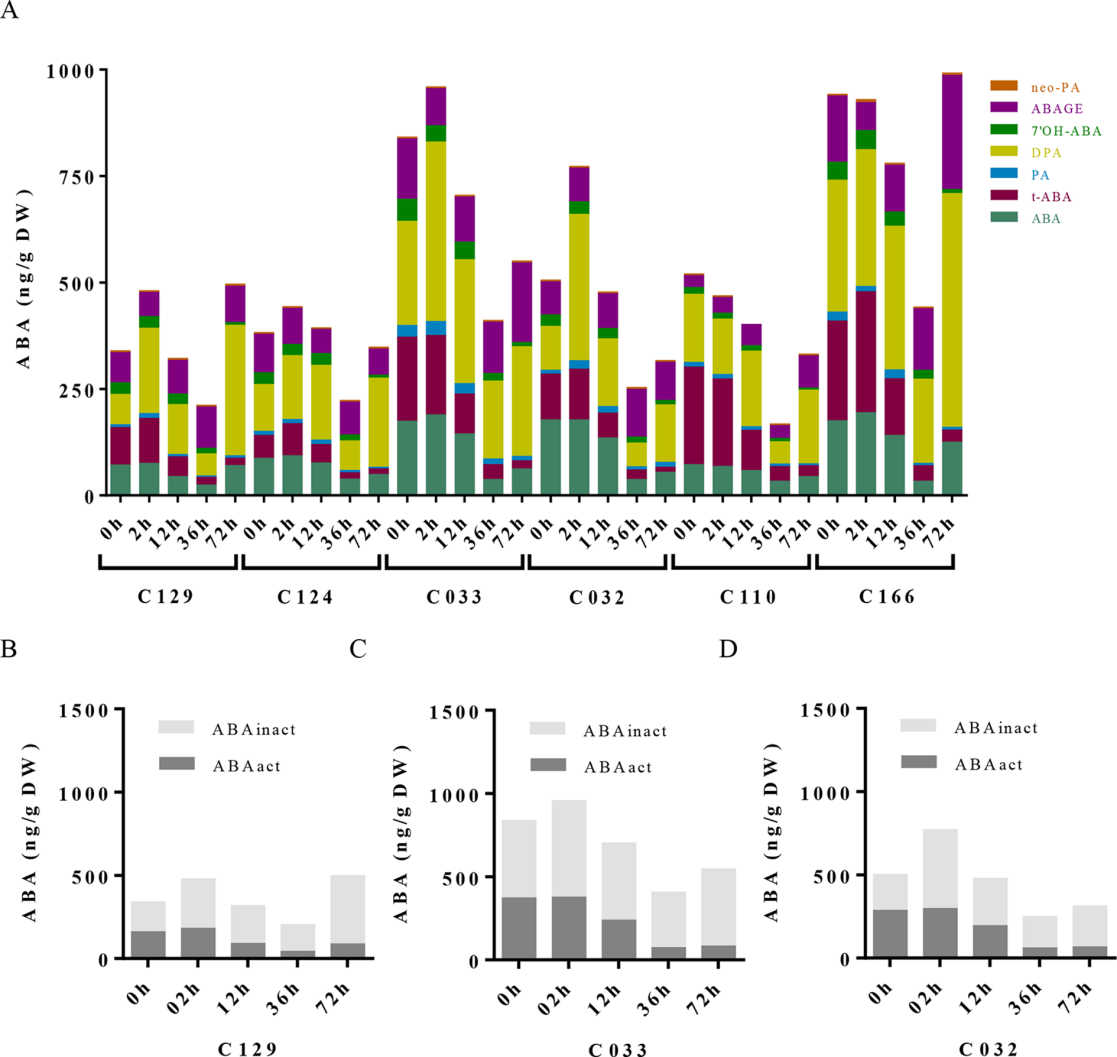
ABA metabolite profile in WOSR accessions has uncovered the contribution of ABA pathway to variability on *B. napus* seed germination dynamics. **(A)** ABA levels progressively diminished along germination in all WOSR accessions, but steady state levels of ABA in dry seeds varied between them. Stacked columns histograms show the distribution of ABA and related metabolites (ABA, PA, DPA, 7′-OH-ABA, neoPA, and ABA-GE) in the WOSR accessions during seed germination. **(B)**, **(C)**, and **(D)** Levels of ABA active (ABA + t-ABA) and inactive ABA metabolites (DPA, ABAGE, PA, 7’OH-ABA, and neo-PA) are higher in C032 and C033 compared to C129, correlating with lower germination rates. Stacked columns histograms show the relative accumulation patterns of ABA active and inactive metabolites in C129, C033, and C032 accessions, respectively. All data were obtained using three pooled biological replicates (50 seeds per replicate). For complete value data set, see **Supplementary Table S1** (**Datasheet 3**).

**Figure 4:**
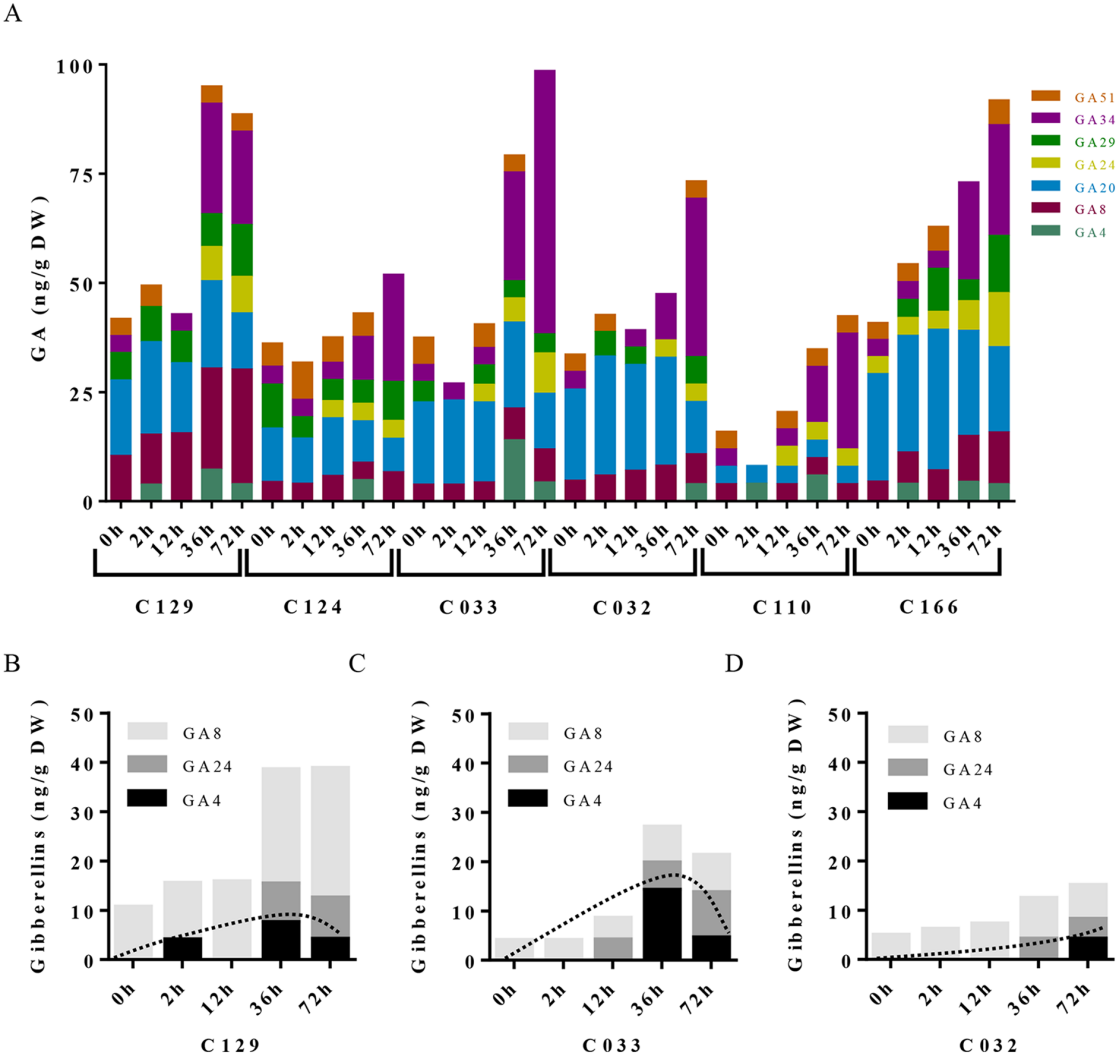
Gibberellins dynamics underlie differential seed germination in WOSR accessions. **(A)** Levels of all GA metabolites differentially increase in WOSR accessions during seed germination. Stacked columns histograms show the distribution of GA metabolites (GA_51_, GA_34_, GA_29_, GA_24_, GA_20_, GA_8_, GA_4_) in the varieties during seed germination. **(B)**, **(C)**, and **(D)** Active gibberellin GA_4_ and GA_24_ accumulation was markedly delayed in C032 accession from 36 to 72 hai compared to C129 and C033, and GA_8_ content is higher in C129 accession compared to C033 and C032, correlating with their germination speed. Stacked columns histograms show the relative accumulation patterns of GA_4_ (active GA), GA_24_ (GA_4_ precursor), and GA_8_ inactive GAs in C129, C033, and C032 accessions, respectively. Dashed curves highlight the GA_4_ (active GA) accumulation dynamics in each accession. All data were obtained using three pooled biological replicates (50 seeds per replicate). For complete value data set, see **Supplementary Table S1** (**Datasheet 3**).

To dissect the influence of hormonal pathways (ABA, auxin, cytokinins, and GAs) in *B. napus* germination, we performed hormone profiling of our six WOSR genotypes at different germination times (**Supplementary Table S1**, **Datasheet 3**). Correlation analyses between GRs and hormone levels along time lead to the identification of significant positive correlations of germination with GAs (GA_4_, GA_8_, GA_24_, and GA_34_) and cytokinins (c-Z and t-Z) levels, whereas significant negative correlations were found with ABA and t-ABA (active ABA species), along with PA and 7´OH-ABA (inactive ABA catabolites) levels. Additionally, negative correlations were identified between ABA and GAs content, confirming their opposite role in *B. napus* germination (**Supplementary Figure 2A**). Consistently, we observed a constant decrease in ABA content very early from imbibition in all the accessions, starting between 2 and 12 hai and reaching its lowest levels between 12 and 36 hai (**Figure 3A**). However, steady state levels of ABA in dry seeds varied between accessions despite grouping in different clusters according to their germination kinetics. C129, C124, and C110 showed lower levels of ABA in dry seeds at early time points (2 and 12 hai) compared with C032, C033, and C116. However, at 36 hai, homogeneous low ABA levels were found in all varieties examined, concordant with germination data described before (**Figures 1A, B**). Consequently, levels of inactive ABA metabolites (DPA, ABAGE, PA, 7’OH-ABA, and neo-PA) were higher in C032 and C033 compared to C129 (**Figures 3B–D**). Together, all of these results suggest that although ABA content is not a major contributor to seed germination variability in *B. napus*, it could affect germination speed in combination with other traits, as we have observed for the C129 accession. In agreement with our correlation analysis, we found in our hormone profile data that GAs increased throughout the germination process with a peak at 36–72 hai in all of the WOSR accessions, coinciding with simultaneously lower levels of ABA (**Figure 4A** and **Supplementary Table S1**, **Datasheet 3**). However, detailed examination of GA content accumulation for each accession at different times after imbibition showed that accumulation of bioactive gibberellin (GA_4_) and GA_24_ was markedly delayed in C032 (between 36 hai and 72 hai) compared to C129 and C033 (between 12 and 36 hai), according to its slower germination speed (**Figures 4B–D**). Interestingly, we also found a significant difference in the GA_8_ content, the inactive catabolite of active GA_1_, being higher in C129 compared with C033 and C032. These results would suggest that in contrast to *Arabidopsis*, where GA_4_ has been described to be the major endogenous active GA in germinating seeds, GA_1_ might also play a key role in WOSR seed germination (Ogawa et al., 2003; Nguyen et al., 2016). Based on these results, we propose that kinetics of GA levels might underlie differential germination in *B. napus*. In addition, we measured IAA levels in all of the WOSR accessions during germination. We found that although we could not establish a significant correlation with GRs from all of the WOSR accessions (**Supplementary Figure 2A**), IAA and IAA-asp levels were higher in C033 and C032 than in C129 (**Supplementary Figures 2B–D** and **Supplementary Table S1**, **Datasheet 3**), suggesting a contribution of these hormones to differences in germination between these accessions. Moreover, C033 showed higher levels of IAA-Asp, a low molecular weight amide conjugate that acts as an intermediate during the auxin degradation pathway (Tam et al., 2002). We have previously shown that the crosstalk of auxin-ABA is important for germination in *B. napus* seed*s* (Liu et al., 2013; Thole et al., 2014; Nguyen et al., 2016). It has been proposed that IAA acts as an enhancer of ABA in germination. Accordingly, we found a positive correlation between IAAs and ABA (**Supplementary Figure 2A**). These results suggest that higher levels of IAA and ABA at early time points could slow down germination speed in C032 and C033 varieties, suggesting that ABA/auxin balance may contribute to the differential germination in *B. napus*.

**Figure 5:**
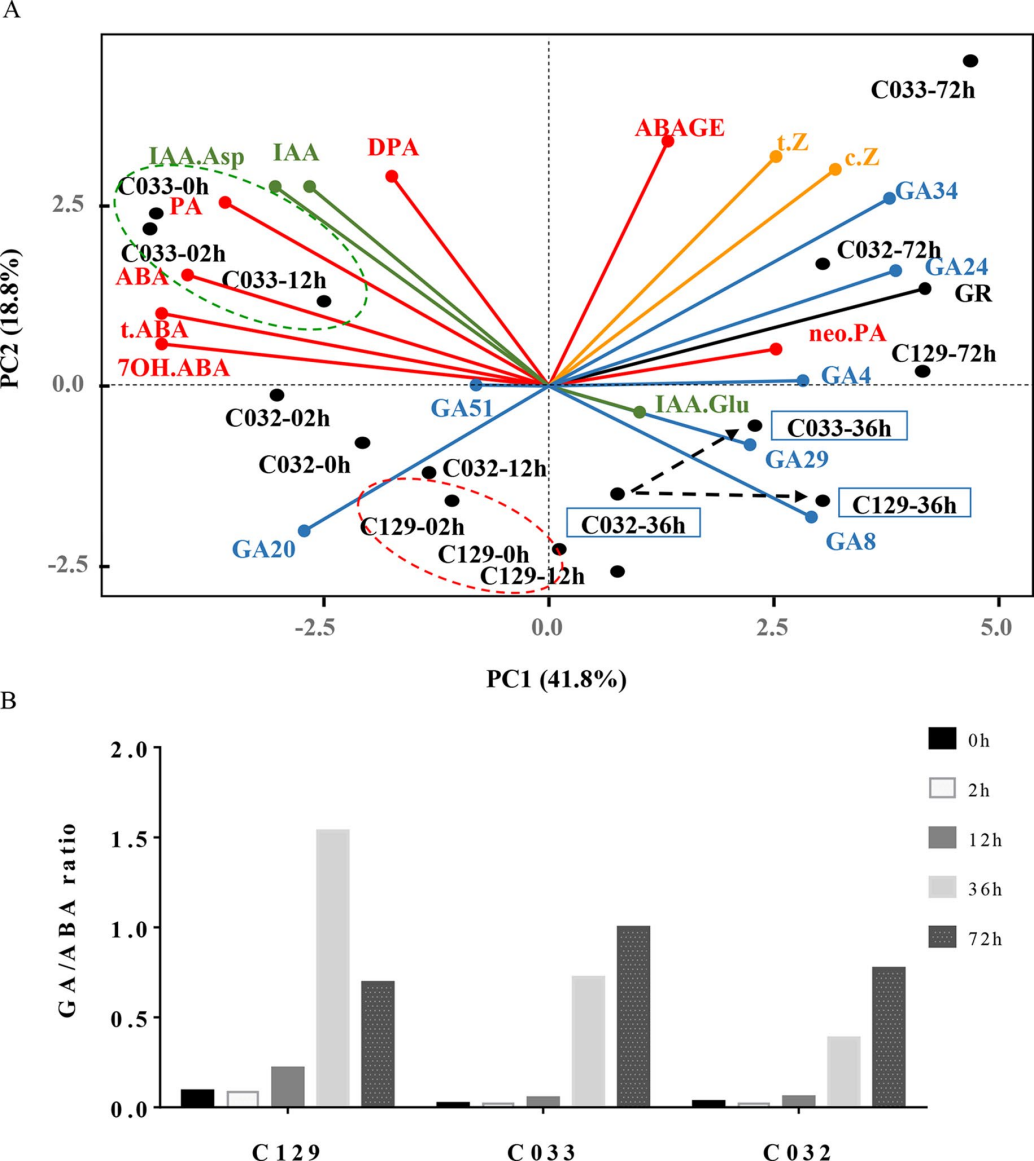
Differences in hormonal dynamics underlie germination variability in WOSR accessions. Differential and combinatorial hormonal levels of ABA, GAs, and IAAs during the germination process contribute to differences in the germination traits observed in WOSR accessions. **(A)** Biplot of principal component analysis (PCA) based on hormone profiles and seed germination rates in the six WOSR accessions. Each hormone class is shown in one color: ABA (red), GAs (blue), auxins (green), and cytokinins (orange). Dashed green circle highlights C033 clustering at early time points (0–12 hai), correlating with higher IAA-Asp levels. Dashed red circle highlights C129 clustering at early time points (0–12 hai), correlating with lower ABA levels. Dashed black arrows highlight the delay in GA accumulation observed in C032 compared to C033 and C129. WOSR accessions are shown in black. **(B)** GA:ABA ratio dynamics of WOSR accessions during seed germination. All data were obtained using three pooled biological replicates (50 seeds per replicate). For complete value data set, see **Supplementary Table S1** (**Datasheet 3**).

All of these results reinforce the hypothesis that the balance between the different hormonal pathways regulating germination is responsible for the differential germination process observed in the studied WOSR accessions. To confirm this, we performed a PCA analysis on hormone levels versus accessions at the different time points. As shown in **Figure 5A**, the accessions did not group in the plot, suggesting that no major differences were present in terms of overall hormone levels. However, the 41.8% of the observed variability explained by PC1 in our samples includes major contributors from ABA and ABA inactive metabolites (t-ABA, 7’OH-AB, PA) as well as GA_4_, GA_24_, and GA_34_ levels, supporting a role of hormonal balance in the variability in germination observed in the WOSR accessions. As expected, germination time points of accessions aligned along the PC1 axis as germination progressed and correlated with GA/ABA ratios (from high GA/ABA to low GA/ABA ratios; **Figure 5B**). Interestingly, C129 and C032 are grouped at early time points of their germination but separated after 36 hai likely because their initially similar BA/GA ratios evolve differently along the germination time course. This confirms hormonal combinations at key time points are drivers of germination in *B. napus* (**Figures 5A, B**). Finally, at early time points (from 0 to 12 hai), accession C033 was slightly different from the others primarily due to higher IAA levels as we have described previously (**Figure 5** and **Supplemental Figures 2B–D****)**. All of these results together reinforce the idea that variability in germination traits in *B. napus* is regulated by the combinatorial contribution of several hormonal pathways.

### Major Transcriptome Changes Occur During *B. Napus* Seed Germination

**Figure 6:**
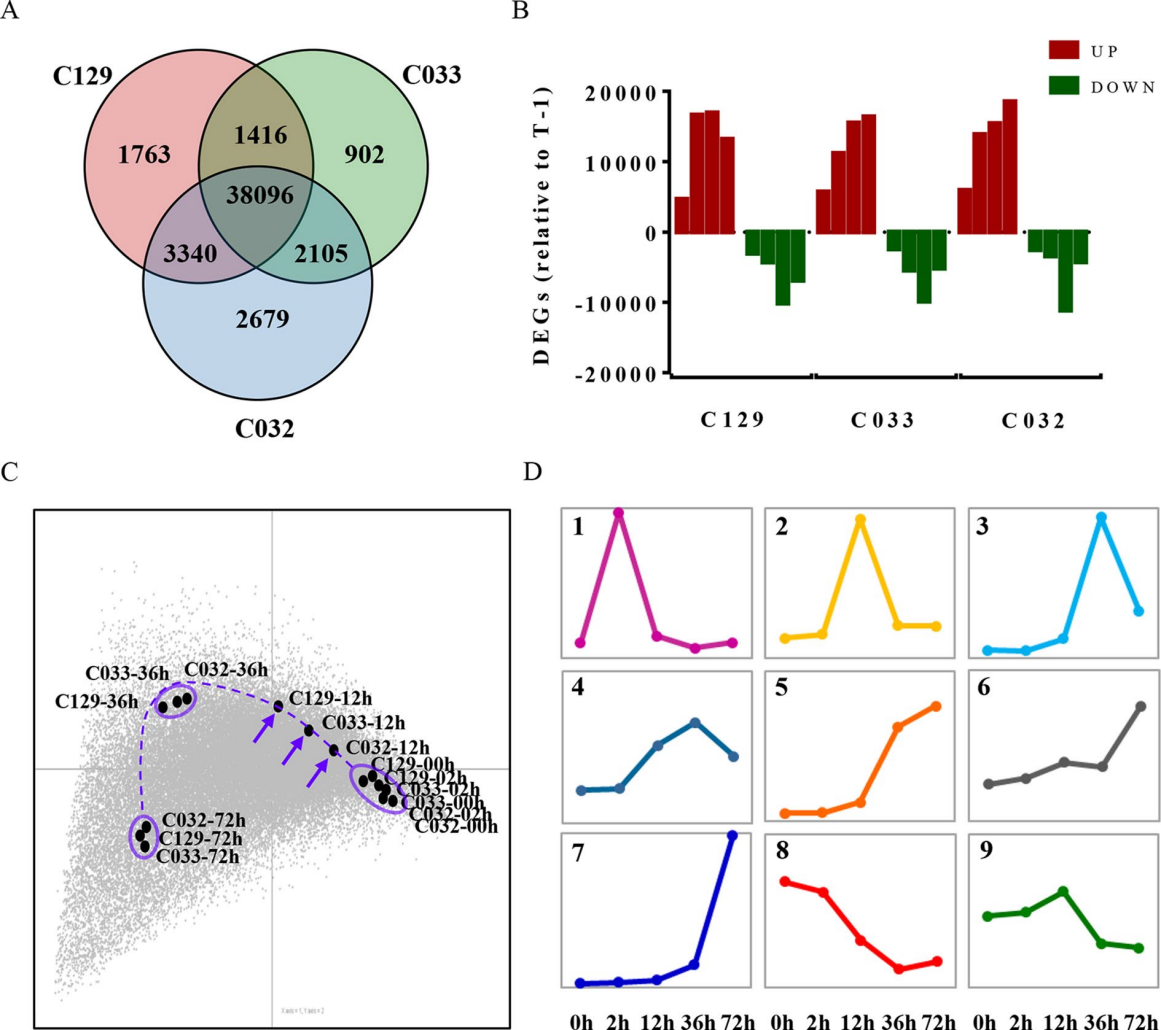
Differential gene expression and transcriptomic dynamics define seed germination in *B. napus* winter accessions. **(A)** Comparison of differentially expressed genes (DEGs) in winter oilseed rape (WOSR) accessions with different germination kinetics showed a common transcriptional regulation. Venn diagram of differentially expressed genes during germination of C129, C032, and C033 seeds from 0 to 72 hai. We compared each time point with 0 hai and also to its previous time point and selected those genes that showed a -1 > log_2_ FC > 1 in at least one of the comparisons. Our final list for C129, C033, and C032 contained 44,615, 42,519, and 46,220 DEGs, respectively. **(B)** Comparison of successive time points for significantly upregulated (red) and downregulated (green) transcripts revealed differences in the timing of significant alterations in the transcriptome. Quantification of the number of DEGs between successive time points during germination in WOSR accessions.T-1 stands for previous time point **(C)** The dynamics of the transcriptional changes associated with the progress of seed germination uncover crucial differences at 12 hai between WOSR accessions. Correspondence analysis between DEGs and WOSR accessions (C129, C033, and C032) during seed germination. DEGs (-1 > log_2_ FC > 1) are represented as a gray dot cloud modeled by the samples (labeled black dots). For each time point, samples tend to lie close to their higher differentially expressed genes, and similar samples tend to stay closer, meaning that they show very similar gene expression profiles. The farther the points are from the origin, the stronger the association between genes and samples. A dashed purple line traces an imaginary trajectory that would represent the dynamics of the transcriptional changes associated with the progress of seed germination. The purple arrows point to the germination time where transcriptional differences between samples are bigger. **(D)** Identification of major clusters representing gene expression dynamics defines a wave of transcriptional activation preceded by an early transcriptional repression during germination of *B. napus*. K-means clustering of differentially expressed genes during C129 seed germination. We use Fuzzy K-means algorithm with Cosine dissimilarity index (1 - Cosine similarity), number of iterations 10, coefficient of fuzziness 1, and initialization random method. The graphs correspond to each of the nine primary clusters obtained (panels 1–9). Each cluster corresponds to a particular gene expression dynamic represented by the colored line. The x-axis represents time after imbibition (2, 12, 36, and 72 hai). Pooled samples of 30 seeds each were used with three biological replicates for each time point.

Germination is a complex trait regulated by the interplay of different physiological, metabolic, and hormonal pathways (Bentsink and Koornneef, 2008; Holdsworth et al., 2008b). To unravel the genetic bases of this complexity, we decided to use comparative transcriptomic analyses of the WOSR accessions during the germination process. Our aim was to use genome-wide transcriptomic analysis of these different genetic backgrounds to identify not only the major transcriptomic changes that define the germination process in *B. napus* but also a set of genes that may contribute to the differences in germination rates detected in these accessions. For this purpose, we carried out RNA sequencing (RNA-seq) on three selected accessions (C129, C032, and C033) at different stages of seed germination. The same time points were used for transcriptomic, metabolic, and hormonal profiling to integrate all of these data into a comprehensive working framework for *B. napus* germination. Our RNA-seq analyses allowed us to detect the expression of 74,019 genes, 73.25% of the total genes in *B. napus* (based on ensemblgenomes, Genome assembly AST_PRJEB5043_v1). Differential gene expression analysis identified 44,615, 46,220, and 42,516 differentially expressed genes (DEGs, -1 > log2FC > 1) in C129, C032, and C033, respectively (**Figure 6A** and **Supplementary Table S2**, **Datasheets 1–3**). More than 60% of detectable genes are therefore changing their expression at the different sampled times. Moreover, comparison between accessions showed that there is an important overlap in DEGs, with approximately 89% being shared between two or more accessions (**Figure 6A**). This finding indicates that the global transcriptome reprogramming that takes place during germination does not change dramatically between WOSR accessions and that a common transcriptional pathway can be defined in *B. napus*. In addition, we observed a clear predominance of upregulated genes over downregulated genes. An increase in the number of upregulated genes reached a maximum between 12 and 36 hai in C129 and at 72 hai in C032 and C033, whereas downregulated genes peaked at 36 hai in all accessions (**Figure 6B**). These results suggest that predominantly a wave of transcriptional gene activation underlies the regulation of germination and that an earlier activation could be responsible for the differences between accessions during *B. napus* seed germination. To explore this possibility, we did correspondence analysis between DEGs and WOSR accessions during the germination process. After projecting the results in a 2D space, we observed again that the transcriptome changes corresponding to each accession were very similar, reinforcing the idea that there are basic genetic and molecular mechanisms governing germination in *B. napu*s (**Figure 6C**). This analysis also highlights that although samples tend to group together at each time point, indicating their similar gene expression profiles, some transcriptomic differences were detected at 12 hai just ahead of the first germination events observed in our germination assays (**Figure 1**). At this time point, C129, C033, and C033 did not cluster together but arrayed on the graph according to their seed germination speed (**Figure 6C**). Based on these results, we could speculate that differences on the transcriptional activation of key genes at early times (12 hai) precedes and marks the differences in germination speed (36 hai) observed between accessions in *B. napus*.

To find these key genes and to capture gene expression dynamics associated with the germination process in *B. napus*, we focused on C129 accession, which showed the highest germination speed, and categorized the DEGs according to their different transcription profiles during seed germination. K-means clustering of normalized expression data led to the identification of nine expression profiles or clusters (**Figure 6D**). Clusters 1, 2, and 3 contained genes with transient expression at 2, 12, and 36 hai, respectively. On the other hand, clusters 4, 5, and 6 contained transcripts whose expression increased gradually during germination. Cluster 7 contained transcripts with a very sharp increase of expression at 72 hai (postgermination, green seedling stage). Finally, clusters 8 and 9 corresponded to transcripts repressed upon seed imbibition (2 hai) or later (12 hai), respectively. The higher number of clusters with dynamics involving mainly transcriptional activation compared to transcriptional repression (seven out of nine) is consistent with our previous observation (**Figure 6B**).

**Figure 7:**
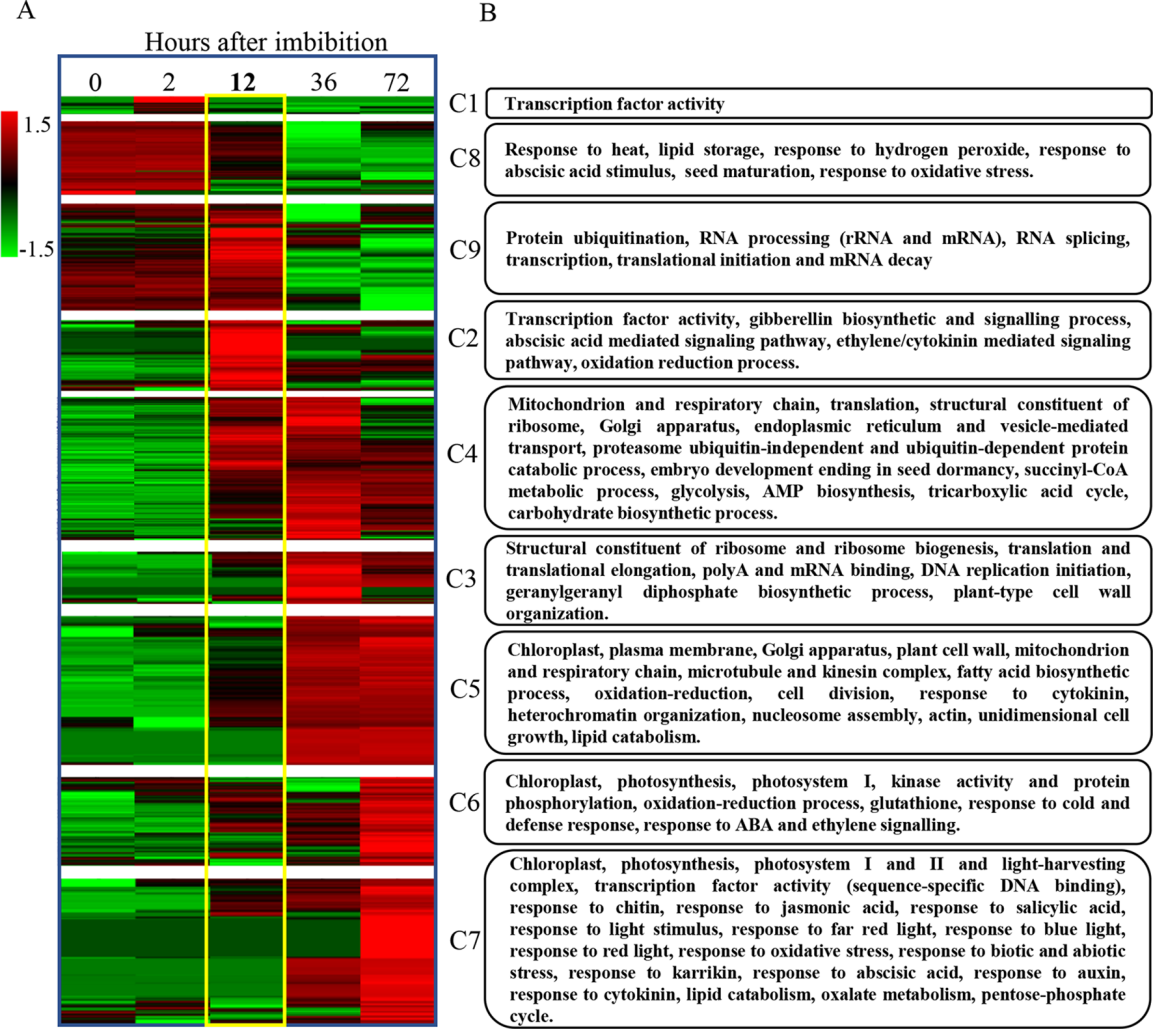
Progressive gene expression and functional gene categorization outline a transcriptomic and genetic framework for seed germination in *B. napus*. **(A)** The identification of the major patterns of gene expression during seed germination highlights 12 hai as a key point for a major switch in transcriptome dynamics. Hierarchical clustering of previously k-means categorized DEGs from C129 WOSR accession and chronological representation of the corresponding heatmaps. Expression values in TPMs for genes included in each gene expression profile, obtained by k-means clustering, were normalized by genes and log transformed, separately. Then, hierarchical clustering using Pearson correlation (complete linkage) and gene leaf order optimization was applied for each cluster (clusters 1–9). The gradient green to red scale represents relative expression units for gene repression or gene induction, respectively. **(B)** We identified the most relevant biological processes being altered during germination in *B. napus*. Functional enrichment for the *B. napus* genes included in each of the previous clusters. The heatmap represents the number of significant functional categories (GO categories) for each cluster obtained by the SEACOMPARE from AgriGO v1.2 (Du et al., 2010) using the *B. napus* GOs and the corresponding p-value obtained with SeqEnrich prediction tool (Becker et al., 2017). The heatmap on the left is a schematic representation of the number of GOs with a p-value < 0.001 cut. P-values > 0.001 are represented as value 1 for graphic simplification. On the right, a summary of some of the most representative functions encompassed in each cluster is presented (see **Supplementary Table S2, Datasheet 14**, for detailed GOs).

After identifying the major patterns of gene expression during seed germination, we performed a hierarchical clustering on each k-means cluster. Later, we represented the corresponding heatmaps in a chronological order based on their expression patterns (**Figure 7A**). The results of these analyses highlighted 12 hai again as a key point for a major switch in transcriptome dynamics. Accordingly, no major transcriptional changes take place before this time point, with most genes showing low expression levels, except the ones categorized in clusters 8 and 9 (repression clusters).

**Figure 8:**
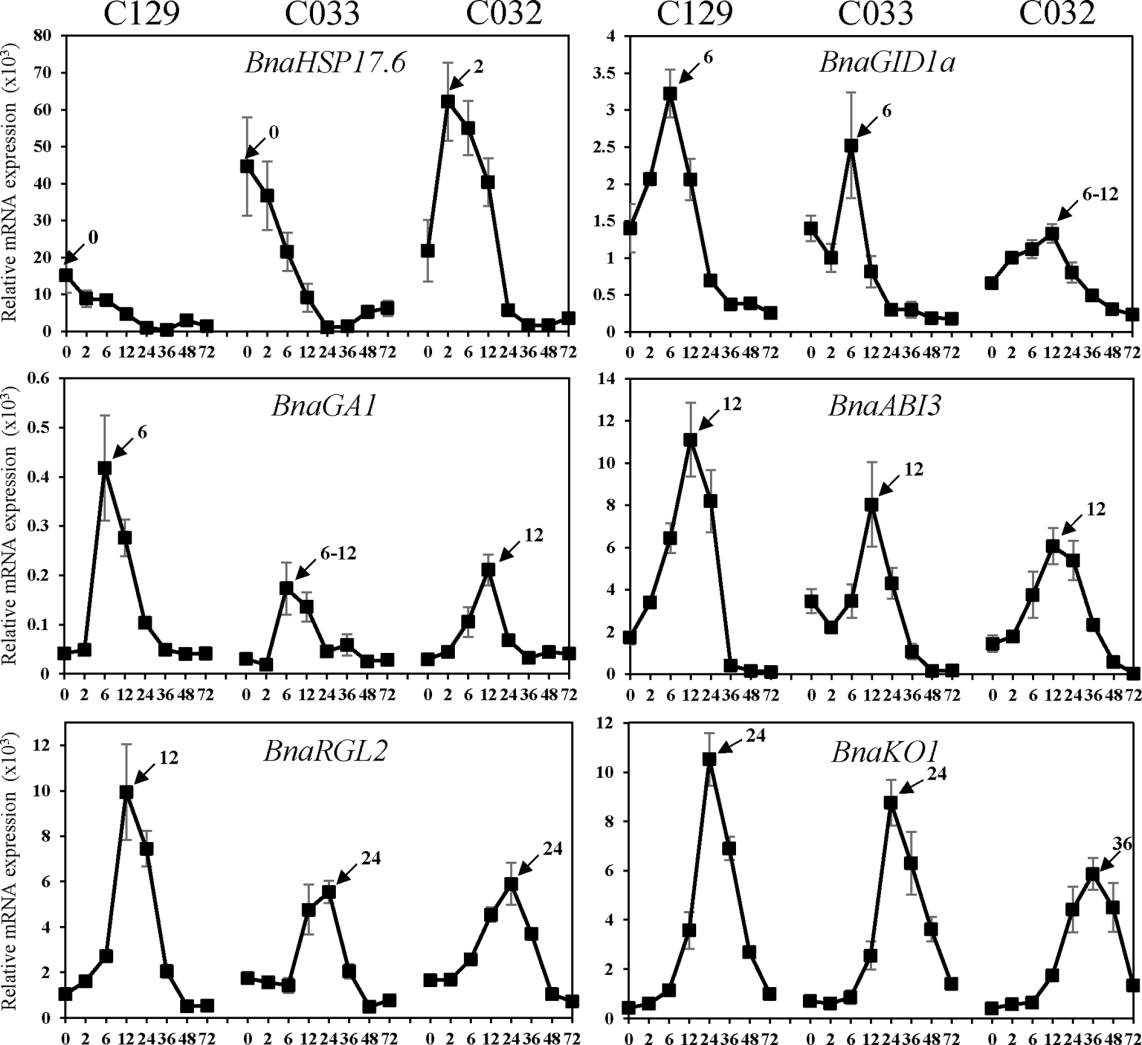
Quantification of *B. napus* gene expression by RT-qPCR during seed germination. RNA was isolated from dry seeds (t = 0) of *B. napus* lines (C129, C033, C032) and at the following time points after seed imbibition: 2, 6, 12, 24, 36, 48, 72 h. Expression values of *B. napus* specific genes were determined by RT-qPCR, and relative expression levels were obtained by normalization with *Bna18S* (*ENSRNA049478716-T1*) rRNA values. Averages and SE of three biological replicates are shown. Amplified gene loci: *BnaHSP17.6* (*BnaC03g05240D/BnaC02g04180D/BnaA03g03740D*), *BnaGID1a* (*BnaC05g46680D*), *BnaGA1* (*BnaA09g01090D*),*BnaABI3 (BnaC03g44820D), BnaRGL2* (BnaC05g47760D), and *BnaKO1* (*BnaC07g28980D*). Three biological replicates were used for each time point and accession.

We carried out a detailed gene ontology analysis of these two clusters to identify the most relevant biological processes being altered at early time points during germination (**Figure 7B** and **Supplementary Table 2**, **Datasheets 6**, **7**). In cluster 8, there is enrichment in genes involved in heat and oxidative stress, seed maturation, seed dormancy, and ABA response, as well as lipid storage. In this cluster, we also found homologs of *Arabidopsis* DNA ligase VI, involved in DNA ligase-mediated rejoining of single- and double-strand breaks (*LIG6*; Waterworth et al., 2016) or homologs of L-isoaspartyl-O-methyltransferase (*PIMT*; Ogé et al., 2008), involved in protein repair as well as chaperones like *BnaHSP17.6*, *BnaHSP22* (**Supplementary Figure S3**; Rueda-Romero et al., 2012), and other chaperones involved in protein folding. These findings agree with the fact that a number of protection and repair mechanisms are set up during seed maturation to keep the integrity of membrane systems, proteins, and DNA during desiccation. Many of these genes are induced by ABA during seed maturation and their expressions only increase upon imbibition if seeds are dormant. A more detailed analysis by RT-qPCR of the kinetics of *BnaHSP17.6* revealed differences in quantity and timing between accessions that correlated with their different germination speeds (**Figure 8**). *ABA1* (Koornneef et al., 1982; **Supplementary Figure S3**) and *NCED6* (Lefebvre et al., 2006), involved in ABA biosynthesis, and *ABI5* (**Supplementary Figure S3**) and *NF-YC9*, among other ABA signaling genes (Koornneef et al., 1982; Finkelstein and Somerville, 1990; Lopez-Molina et al., 2002; Liu et al., 2016), were also present in cluster 8. It is worth to mention that ABA1 catalyzes the first step in the biosynthesis of ABA, and *aba1* mutants have enhanced seed germination (Koornneef et al., 1982; Barrero et al., 2005). ABI5 is a repressor of seed germination able to reactivate late embryogenesis programs (Lopez-Molina et al., 2002). This function requires the presence of ABA to promote ABI5 phosphorylation and protein activity (Piskurewicz et al., 2008), as well as the NF-YC9 transcription factor to support ABI5 transcription (Liu et al., 2016). During seed maturation, ABA accumulates and exerts an inhibitory effect on mechanisms that, otherwise, would trigger germination of developing seeds in the mother plant (preharvest sprouting). Therefore, cluster 8 reflects the shutdown of ABA biosynthesis and signaling pathways necessary to alleviate the inhibition of the germination program. Finally, oleosin family genes were highly abundant in this cluster, reflecting the active accumulation of oil seed bodies during the late seed maturation program. Seed maturation is completed when storage compounds have accumulated, water content has decreased, ABA levels have increased, and desiccation tolerance is established. Thus, genes belonging to cluster 8 were related with the recapitulation of the seed maturation program ending in seed dormancy and the later shift to a germination-driven program of development.

Cluster 9 is the other cluster containing genes with a high transcript accumulation in dry seeds but, in contrast to cluster 8, those transcripts continued increasing their levels until 12 hai and are repressed by 36 hai (coinciding with the transition from germination to greening). Cluster 9 mainly contains genes involved in RNA processing (rRNA and mRNA), translation initiation and elongation, transcription, mRNA splicing, mRNA decay, and protein modification, especially mono and polyubiquitination as well as protein dephosphorylation and degradation. Several genes involved in those processes have been shown to reduce ABA sensitivity during seed germination like *AHG1* (**Supplementary Figure S3**) or *DCAF, HUB1, PRT6*, and *ATE2* (Liu et al., 2007; Nishimura et al., 2007; Holman et al., 2009; Seo et al., 2014). Additionally, cluster 9 is enriched in DEAD-box RNA helicases as well as translation initiation and elongation factors (*eIF4E*, *eIF4G*, Lellis et al., 2010; Dinkova et al., 2011) with possible roles in mRNA selective translation of proteins from seed stored mRNAs that would support germination (Sajeev et al., 2019). Cluster 9 is also enriched in mRNA decapping and decay related genes (*DCP1*, Basbouss-Serhal et al., 2017), especially nonsense-mediated mRNA decay. Selective mRNA decay could also play a role in alleviating dormancy and promoting germination (Basbouss-Serhal et al., 2017). In this cluster, we also found *GID1a*, a GA receptor, suggesting that an increase in GA sensitivity parallels the reduction in ABA sensitivity to promote germination. To study in more detail this gene, we carried out a more comprehensive analysis of its RNA kinetics by qPCR. *BnaGID1a* showed different kinetics between accessions (**Figure 8**). C129 was the only accession with a clear induction upon imbibition peaking at 6 hai and showing a sharp decrease afterward. On the contrary, only a small increase in transcript abundance was detected in C033 and C032 with a sharp decrease afterward, and overall levels were lower than those observed in C129. Additionally, *BnaGID1a* mRNA levels peaked later in C032 when compared with the other two accessions. These results suggest that C129 may be more sensitive to GAs upon imbibition than the other accessions. Interestingly, this cluster also contains *PIL5* (**Supplementary Figure S3**), a negative regulator of seed germination involved in the control of hormone metabolism and signaling (Oh et al., 2004).

The remaining clusters encompass transcriptional activation events taking place during germination. Cluster 2 marks early transcriptional induction events occurring at 12 hai. In this cluster, we found a clear enrichment of genes with transcription factor activity as well as genes involved in GA metabolism and signaling. These GO categories (GO:0009686, GO:0009740, GO:0009739, and GO:0045544) constitute 3.6% of the genes, whereas they only account for 0.75% of all *B. napus* genes (**Supplementary Table S2**, **Datasheet 8**). We also found a significant enrichment of genes involved in ABA perception and signaling as well as in ABA degradation (Vishal and Kumar, 2018). Thus, these results suggest that the previously described changes from low GA/ABA ratios to high GA/ABA ratios (**Figure 5B****)** that will determine the initiation of the germination process in *B. napus* are preceded by transcriptional regulatory events set up mainly at 12 hai. In this context, the presence of *GA1* in this cluster. GA1 catalyzes the first committed step in the GA biosynthesis pathway, and *ga1* mutants only germinate when exogenous GAs are added (Sun and Kamiya, 1994; Ogawa et al., 2003). Expression analyses by qPCR showed that *BnaGA1* mRNA levels peaked later in C032 when compared with the other two accessions, a result similar to that obtained for *BnaGID1a* (**Figure 8**). ABI3 and RGL2 are two important negative regulators of seed germination also present in this cluster. Both regulators are intimately connected in the repression of seed germination by quickly responding to changes in GA/ABA ratios (Piskurewicz et al., 2009). Our qPCR results revealed that *BnaABI3* RNA levels peak at 12 hai in all accessions, while *BnaRGL2* RNA levels peak earlier in the C129 accession (12 hai) compared to C033 and C032 (**Figure 8**). Clusters 3 and 4 included transient and gradual increases of transcriptional activation peaking at 36 hai. Cluster 4 encompassed genes coding for mitochondrion and respiratory chain components, genes related to ribosomes and translation, Golgi apparatus, endoplasmic reticulum, and vesicle mediated transport. We also found genes encoding for key enzymes involved in fatty acid beta-oxidation and glyoxylate cycle (MLS, ICL, Comai et al., 2007), tricarboxylic acid cycle (ACO3, CSY3, Pracharoenwattana et al., 2005; Hooks et al., 2014), carbohydrate biosynthesis, and glycolysis (HXK1, Aguilera-Alvarado et al., 2019). In the case of *BnaHKX1*, we found that the peaking time point in C033 and C032 was delayed compared to C129 (**Supplementary Figure S3**). These results suggest that the main metabolic pathways are fully functional at 36 hai, processing the seed reserves and supplying the embryo with the energy and all of the organic compounds required for its transition to a seedling.

In cluster 3 (genes transiently induced at 36 hai), we found a significant enrichment of genes encoding for structural ribosome constituents as well as genes involved in ribosome biogenesis, translation, and mRNA binding. This suggests that after the germination program has been established, major efforts are dedicated to the translation of mRNAs to protein. The ent-kaurene synthase (KO1), an enzyme required for the GA biosynthesis pathway (Rademacher, 1989; Nambara et al., 2008) is also present in this cluster together with well-known GA-responsive genes, such as *CP1*, *EXPA2*, and *EXPA8* (**Supplementary Figure S3**; Ogawa et al., 2003; Rombolá-Caldentey et al., 2014; Sánchez-Montesino et al., 2019). *KO1* is a useful marker for GA levels given that its transcription is under feedback regulation, stimulated by paclobutrazol and repressed by GA_4_ (Ogawa et al., 2003; Nambara and Marion-Poll, 2005). Consequently, we observed that RNA levels of *BnKO1* increase upon imbibition at 12 hai and peak at 24 hai for C129 and C033 and at 36 hai for C032 (**Figure 8**). These transcriptional profiles are in agreement with the germination kinetics of our accessions (**Figure 1A**) as well as with their timing of GA_4_ accumulation (**Figures 4B–D**). The sharp decrease of RNA levels observed after their peaking times may indicate that this gene, as in *Arabidopsis*, is also under GA negative feedback in *B. napus*.

Cluster 5 contains genes that are mainly induced at 36 hai and maintained at high expression levels until 72 hai. This cluster was enriched in genes involved in cell wall biogenesis and organization (*XTH9*, **Supplementary Figure S3**; Shin et al., 2006; Sánchez-Montesino et al., 2019), fatty acid biosynthesis (*FDH/KCS10*, **Supplementary Figure S3**; Yephremov et al., 1999; Rombolá-Caldentey et al., 2014), heterochromatin organization, and nucleosome assembly (specially *H2A*-related genes). Interestingly, we also found a significant enrichment in genes related with microtubule and kinesin complex, mainly involved in cytokinesis, and many cell division and cell cycle-associated genes. TCP14, a transcription factor that positively regulates seed germination by mediating GA action and cyclin gene expression, was also found in this cluster (**Supplementary Figure S3**; Tatematsu et al., 2008; Resentini et al., 2015). After embryo formation in the dry seed, developmental control imposes a pause in cell proliferation. However, imbibition does not cause an immediate entry into the cell cycle (Vázquez-Ramos and de la Paz Sánchez, 2003). In general, cell division is concomitant with or take place after radicle protrusion (Barrôco et al., 2013), and cell proliferation is an absolute requirement for seedling establishment (Vázquez-Ramos and de la Paz Sánchez, 2003). Therefore, the enrichment in cell wall biogenesis and organization and cell cycle genes suggested that the transition from germination (embryo expansion) to post-germinative (seedling) growth in *B. napus* takes place at around 36 hai, agreeing with our previous phenotypic, metabolic, and hormonal data, pointing to this time as crucial for germination performance. Finally, both clusters 6 and 7 contain induced genes with maximum expression at 72 hai but differing in their activation dynamics. They are highly enriched in genes associated with photosynthesis (photosystems I and II and light-harvesting complex). Additionally, cluster 7 was highly enriched in genes associated with response to both biotic and abiotic stresses as well as in light perception (red, far red, and blue light) and hormone signaling, specially abscisic acid, auxins, ethylene, and cytokinins. These GOs enrichment indicates that the switch to a photosynthetic seedling is taking place at this time point, marking the completion of the germination process and the transition to the seedling establishment phase.

All of these results together define a transcriptional framework determined by a wave of transcriptional activation preceded by an early transcriptional repression that regulates specific biological process during germination in *B. napus*. Genes associated to key regulatory events identified in this analysis could be used as genetic markers as well as targets for improvement of *B. napus* germination.

### Key Protein Interactions Involved in ABA/GA Signaling and Crosstalk During Germination Are Conserved in *B. napus*

Several regulatory proteins have been shown to play important roles in ABA/GA signaling and crosstalk during seed germination in *Arabidopsis thaliana* and other plant species. In particular, direct protein–protein interactions between some of these key regulators have been found to be essential for a coordinated response to both hormones. We used a yeast two-hybrid system (Y2H) to test whether these strategic interactions have been conserved in *B. napus*.

**Figure 9:**
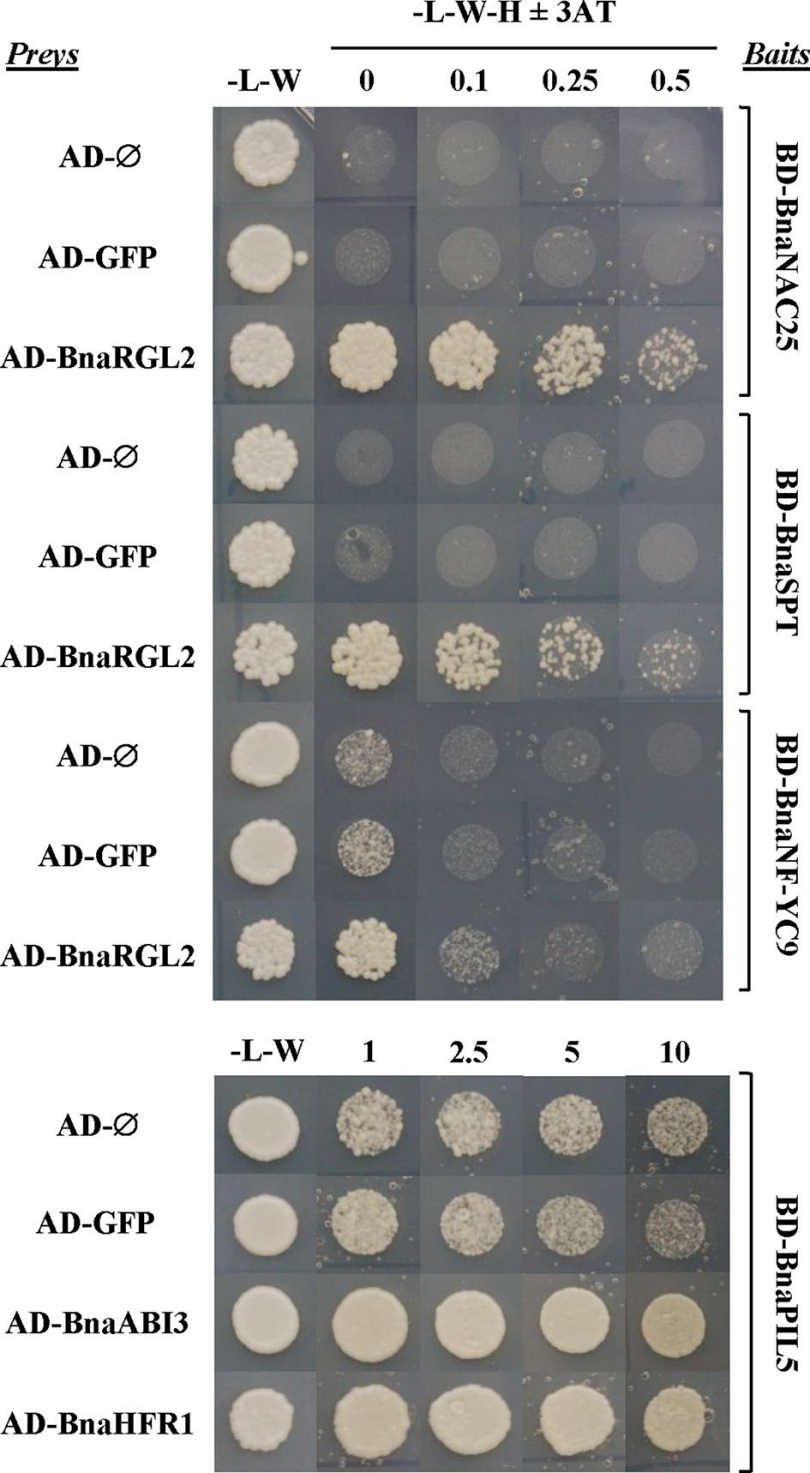
Protein interactions between important regulators of seed germination are conserved in *B. napus*. Interactions between selected *B. napus* transcription factor (TF) proteins analyzed in a yeast two-hybrid system. Bait constructs were generated by N-terminal translational fusions between the GAL4-binding domain (BD) coding sequence (CDS) and selected TF CDSs (*BD-BnaNAC25*, *BD-BnaSPT*, *BD-BnaNF-YC9*, and *BD-BnaPIL5*). Prey constructs were generated by N-terminal translational fusions between the GAL4-activation domain (AD) CDS and selected TF CDSs (*AD-BnaRGL2*, *AD-BnaABI3*, and *AD-BnaHFR1*). The GAL4-AD and the GAL4-AD fused to the CDS of the green fluorescent protein (GFP) were used as negative controls. Yeast strains containing baits were mated to strains containing preys in the combinations indicated in the figure, and the diploid cells obtained were grown on diploid (-L-W) and screening (-L-W-H) plates with or without 3-AT. Cells containing combinations of baits and preys able to interact were able to grow on screening plates at concentrations of 3-AT that blocked the growth of negative controls.

The RGL2 protein is the main DELLA protein involved in the control of seed germination (Piskurewicz et al., 2008). RGL2 is able to interact with NF-YC transcription factors to promote transcription of *ABI5* to negatively regulate seed germination (Liu et al., 2016). *BnaRGL2* kinetics (**Figure 8**), as well as *BnaNF-YC9* and *BnaABI5* (**Supplementary Figure S3**), are similar to their *Arabidopsis* counterparts, suggesting that their roles could be conserved. We tested if their interactions are also conserved using Y2H. As observed in **Figure 9**, yeast cells carrying *AD-BnaRGL2* with the *BD-BnaNF-YC9* constructs show enhanced growth in the presence of increasing concentrations of 3-aminotriazole when compared to cells carrying the *AD-BnaRGL2* and any of the negative control plasmids (*AD-Ø*, *AD-GFP*). These results indicate that BnaRGL2 is able to interact with BnaNF-YC9 as in *Arabidopsis*. RGL2 also sequesters NAC25 in a physical interaction to repress endosperm cell expansion, cell wall-related gene expression, and seed germination (Sánchez-Montesino et al., 2019). We observed that *BnaEXPA2*, one of the main targets of the RGL2-NAC25 complex, was expressed as *AtEXPA2* and that both BnaRGL2 and BnaNAC25 proteins are able to interact (**Figure 9**). *SPT* is another negative regulator of seed germination and cotyledon expansion that has been shown to interact with RGL2 in yeast (Penfield et al., 2005; Gallego-Bartolomé et al., 2010). Yeast cells carrying *AD-BnaRGL2* with *BD-BnaSPT* constructs also show growth in selective media, indicating a similar interaction than in *Arabidopsis*. All of these results indicate that BnaRGL2 interactions with key regulators of germination are conserved in *B. napus*.

PIL5 is another negative regulator of seed germination, integrating light and hormone signals (Oh et al., 2004; Oh et al., 2006; Oh et al., 2009). When PIL5 protein is not degraded by light, their regulatory properties depend on several interactions with other proteins and gene promoters. Moreover, PIL5 and ABI3 collaboratively activate the expression of *SOM* by directly binding to and interacting with each other at the *SOM* promoter (Park et al., 2011). In turn, SOM negatively regulates seed germination by modifying the expression levels of ABA/GA metabolic genes (Kim et al., 2008). The kinetics of these genes (**Figure 8** and **Supplementary Figure 3**) are similar to those observed for *Arabidopsis* genes (Parcy et al., 2007; Piskurewicz et al., 2008; Park et al., 2011; Hsieh et al., 2012), suggesting that this important regulatory network is conserved in *B. napus*. Indeed, the result from our Y2H experiment indicates that BnaPIL5 (*BD-BnaPIL5*) and BnaABI3 (*AD-BnaABI3*) proteins are able to interact strongly in this system (**Figure 9**). Contrary to PIL5, HFR1 is a positive regulator of light-induced seed germination able to directly interact with and sequester PIL5 to prevent it from binding to its target genes (Shi et al., 2013). In addition, this interaction promotes reciprocal codegradation of PIL5 and HFR1 (Xu et al., 2017). This way, light-induced germination promotes increased abundance of HFR1, thus enhancing PIL5 degradation and leading to increased GA levels (Oh et al., 2006; Xu et al., 2017). As observed in **Figure 9**, BnaPIL5 and BnaHFR1 are able to interact strongly since yeast cells carrying the *BD-BnaPIL5* and AD-*BnaHFR1* constructs have enhanced growth in the presence of 3-AT above control levels and as strongly as the BnaPIL5-BnaABI3 interaction.

Altogether, these results suggest that protein interactions between transcription factors with a relevant role in seed germination by modifying and signaling hormone levels have been conserved in *B. napus*.

## Discussion

Seed germination is a major component of seed vigor affecting plant survival and crop yield. Our analyses of a panel of *B. napus* accessions showing robust differences in seed germination have determined that the speed of this process is responsible for germination variability. Briefly, despite our accessions being very similar in terms of seed viability (understood as the germination potential of the seed, final germination rates), major differences in seed germination were observed in T50 values, spanning 15 h between the fastest (C129) and slowest (C032). In other words, when considering 36 hai as the average T50, a 30% GR difference between extreme accessions was observed. Although seedling establishment is largely dependent on seed viability, our results indicate that high seed viability does not always correlate with high germination speed in *B. napus*. Given that germination speed could have a greater impact on seedling survival and crop yield when environmental conditions are not favorable, this trait will be a valuable parameter to estimate and to improve seedling establishment in all environmental conditions affecting this crop in the field. In fact, farmers require vigorous seeds that ensure the reliable and successful establishment of their crops under environments subjected to changing environmental cues (Finch-Savage and Bassel, 2016). Thus, seeds performing well under optimal conditions are also more likely to perform better under stress (Bettey et al., 2000; Foolad et al., 2007). It has been shown that *B. napus* seed germination can be substantially improved (Hatzig et al., 2015), and our results suggest that using the genetic variability to increase germination speed is a promising approach to achieve it. Moreover, our results have confirmed that OSR seeds germinate faster than *Arabidopsis* seeds, in agreement with previously published data (Li et al., 2005; Hatzig et al., 2015a; Kubala et al., 2015; Wang et al., 2016; Gu et al., 2019; Luo et al., 2019). This acceleration of germination in *B. napus* could be reflecting the enhanced pressure to germinate that crop seeds have to face on agricultural challenging environments, again pointing to this trait as an interesting target to increase *B. napus* seed performance in the field. However, testing this hypothesis is not easy given the existing differences in seed size between *Arabidopsis* and oilseed rape and the absence of *B. napus* wild-type accessions.

Seed reserves, such as oils, proteins, and carbohydrates, are key for metabolism activation during seed imbibition along the germination process and serve to support early seedling growth (Weitbrecht et al., 2011; Nonogaki, 2014; Paszkiewicz et al., 2017). Our metabolic profiles agree with other seed germination profiles in other plants and crops, where at later stages of germination, there is also an increase in sugars (fructose, glucose, and maltose) and minor carbohydrate metabolism (raffinose, sorbitol, and xylose) related with the increased necessity of energy during the germination process as well as compounds associated with cell wall metabolism (myo-inositol and galactinol) (Howell et al., 2008; Gu et al., 2016). During seed germination, carbon skeletons of oil reserves are utilized to produce sugars to feed the energy metabolism machinery (glycolysis, Krebs cycle, oxidative phosphorylation, and mitochondrial electron transport)—thanks to the glyoxylate cycle. Interestingly, PCA analysis of carbohydrates evidenced that the high germination speed C129 accession has significant lower levels of malate at all time points analyzed (**Figure 2C**). Malate is an important metabolite that can be oxidized to oxaloacetate, a precursor of gluconeogenesis, and is produced by the glyoxylate cycle (glyoxysomes) as well as by the Krebs cycle in the mitochondria (Eastmond and Graham, 2001). One explanation for our result would be that C129 is able to use malate faster than other accessions, resulting in lower basal levels of the metabolite. Consequently, this accession would be more efficient generating energy required for germination that, in turn, will be accelerated. Although our analyses on amino acid levels yielded results similar to those obtained for *Arabidopsis* (**Figure 2B**; Fait et al., 2006), a peak of aspartate at 36 hai was observed for C129 but not for C033 and C032. The plant Asp-family pathway leads to the synthesis of the essential amino acids lysine, threonine, methionine, and isoleucine and, for this reason, has been of interest for plant breeders (Jander and Joshi, 2009; Galili, 2011). Besides their importance for human food quality, these amino acids are also well-known donor metabolites that feed the TCA cycle (Weitbrecht et al., 2011). Thus, higher levels of aspartate could also be related to extra carbon being available for germination and early seedling development. Both results suggest that malate and aspartate would be good markers to monitor germination efficiency in this crop.

ABA accumulates in dry seeds during seed and embryo development, but endogenous ABA content decreased significantly within 6–24 h after the onset of imbibition in *Arabidopsis* and rice (Ali-Rachedi et al., 2004; Ye et al., 2012). Consistently, ABA levels progressively diminished over time in all accessions, although steady state levels of ABA in dry seeds varied between them. Thus, we found that among the accessions studied, rapidly germinating C129 has lower levels of ABA in dry seeds and at early time points. However, our results combining germination kinetics of C129, C033, and C032 and clustering analyses indicate that ABA content does not seem to be a major contributor to germination variability on *B. napus* seeds. Moreover, RNA levels of key genes involved in ABA metabolism are similar between the accessions along the sampled germination times, resembling the pattern observed for their orthologs in *Arabidopsis* (**Supplementary Figure S3**). However, it seems that is the ABA/GA balance and the sensitivity to these hormones that play a central role in the control of germination (Finch-Savage and Footitt, 2017). Bioactive GA accumulates in the embryo just before radicle protrusion (Ogawa et al., 2003; Kucera et al., 2005). We detected GA_4_ (bioactive) in the C129 and C033 accessions and found that its accumulation is delayed in the C032 accession (selected for its slow germination speed). In accordance with these results, we found that the kinetics of two genes encoding key enzymes for early steps of the GA biosynthetic pathway (*BnaGA1* and *BnaKO1*) are delayed in the C032 accession in comparison to C129 and C033 (**Figure 8**). Taking all these data together, the ABA/GA balance could explain some of the differences observed in germination speed between accessions. Thus, C129 has low ABA and medium GA levels, C033 has more ABA but higher GA levels, and C032 has high ABA (similar to C033) but delayed accumulation and low levels of GA. In addition, it has been proposed that IAA acts as an enhancer of ABA in germination (Liu et al., 2007; Nguyen et al., 2016). Interestingly, we detected high levels of IAA at early time points in C032 and C033 varieties (**Supplementary Figure S2**) that could be accounting for slowing down germination speed. Moreover, the higher IAA-Asp levels observed in the C033 accession compared to C032 suggest that the former would be able to overcome the inhibitory effects of IAA by activation of IAA degradation pathways to ensure germination (Ljung, 2013).

Unexpectedly, we also found significant differences in GA_8_ content, being higher in C129 accession compared with C033 and C032. GA_8_ is an inactive GA catabolite from the early 13-hydroxylation pathway where active GA_1_ is formed (Hedden and Phillips, 2000; Sun, 2008). The amount of GA_1_ is usually 10% of that detected for GA_4_ (Yamauchi et al., 2004), and our inability to detect GA_1_ could be due to the existence of lower levels compared to other species. Although GA_4_ is likely to be the major endogenous active GA in *Arabidopsis* (Talon et al., 1990; Derkx et al., 1994; Hedden and Kamiya, 2002; Ogawa et al., 2003), our results suggest that GA_1_ may play a key role in WOSR seed germination. In *Arabidopsis*, it has been shown that the effects of GAs on seed germination are also related to the sensitivity to the hormone (Nakajima et al., 2006). Among the three *Arabidopsis* GA receptors, genetic evidence has shown that GID1a and GID1c are the most important for seed germination (Voegele et al., 2011) and that GID1a is the most abundant (Cao et al., 2006; Griffiths et al., 2006; Hauvermale et al., 2014). In that respect, it is worth mentioning that C129 is the accession with the highest levels of *BnaGID1a* mRNA (**Figure 8**), and the sharp decrease in RNA levels of *BnaGID1a* observed in the accessions, that preceded peaking levels of GA_4_, is compatible with its role as GA receptor (Griffiths et al., 2006). Thus, we propose that *B. napus* accessions differences in GA homeostasis and GA sensitivity underlie variation in germination speed in *B. napus* accessions.

Transcriptomic analyses are extremely useful to uncover the basic biological process underlying any trait. Correspondence analysis between DEGs and WOSR varieties along the germination process and their projection in a 2D space highlighted that changes in the transcriptomes corresponding to each accession were very similar (89%; **Figure 6C**). In fact, most of the RNA kinetics (RNA-seq or/and qPCR) of genes related to ABA and GA metabolism and signaling, including key regulators of the process, had similar patterns of gene expression between accessions. In the same way, important protein–protein interactions known to control germination by integrating environmental and hormone signals in other species (Gallego-Bartolomé et al., 2010; Park et al., 2011; Liu et al., 2016; Xu et al., 2017; Sánchez-Montesino et al., 2019) were found to be conserved when using *B. napus* ortholog proteins (**Figure 9**). All together, these results reinforce the idea that the basic molecular mechanisms governing germination are the same in the different accessions. However, some transcriptomic differences were located mainly at 12 hai, where C129, C033, and C032 did not cluster together along transcription progress associated with seed germination. Indeed, several genes were found to have different kinetics between the accessions that could contribute to their distinct germination speed (**Figure 8**). Most of these differences fits with an early activation of germination-related gene networks, mainly related with hormonal pathways, in the rapid-speed germination variety C129 and a delayed response of slow-speed C032 (**Figure 8** and **Supplementary Figure S3**). Furthermore, some of these genes could contribute directly to some of the seed germination traits differences in WOS. Thus, we found that some genes previously associated to seed germination, *BnaTPC1* and *BnaGDSL1*, showed differential expression kinetics between our accessions (**Supplementary Figure 3**; Hatzig et al., 2018; Ding et al., 2019). *BnaGDSL1* is highly induced during seed germination and is phylogenetically very close to *BnaLIP1*. The *Arabidopsis* orthologue gene of *BnaLIP1* is a marker gene for GA-induced expression during seed germination in the embryo epidermis, positively affecting seed germination speed (Rombolá-Caldentey et al., 2014). Seed germination requires GA signaling in the epidermis mediated by DELLA and HD-ZIP IV proteins that control the expression of genes involved in growth of the embryonic axis (Rombolá-Caldentey et al., 2014). One of the target genes of this regulatory complex is *EXPA8* (cluster 3; **Supplementary Figure 3**), an important player for cell wall remodeling during growth and a convergence point for several signaling pathways (de Lucas et al., 2008; Feng et al., 2008; Stavang et al., 2009; Bai et al., 2012; Gallego-Bartolomé et al., 2012; Park et al., 2013). Interestingly, this gene was found highly expressed in C129 compared to C032, and C033, supporting again a GA-related mechanism underlying germination speed in these varieties. We have seen a correlation between ES of the radicle and germination speed parameters. Taking into account that root elongation is mediated by cell expansion, it is intriguing to think that a GA-mediated cell expansion could be taking part in this germination response. In summary, our results provide a comprehensive framework of seed germination dynamics in OSR. Metabolic, hormonal, and transcriptomic data of this study could be used not only to evaluate germination performance of *B. napus* accessions but also to identify key players for their application in biotechnological and breeding programs in this crop. Although further analyses with biparental segregation populations will be needed to validate these molecular signatures for seed germination, in this study, we have identified a combination of metabolites, hormones, and genes (aspartate, malate, IAA/IAA-asp, GA/ABA, *BnaGA1*, *BnaKO1*, *BnaGID1a*, *BnaEXPA8*) that correlate with germination performance and could be putative targets for crop improvement of *B. napus*.

## Data Availability Statement

This manuscript contains previously unpublished data. The name of the repository and accession number(s) are not available.

## Author Contributions

MB carried out *in silico* data analysis and together with MP and LO-S interpreted the results. GW generated the RNA-seq sequence data and prepared samples for additional analyses. SH was involved in generation of the metabolite/hormone data. JC-C did RT-qPCRs, germination experiments, and seed measurements, and GC-C carried out protein–protein interactions assays. NN and RS coordinated previous experiments to select appropriate *B. napus* genotypes. LL and GB performed the metabolite profiling assays. NN, AB, LL, and GB analyzed the metabolite data. MB, MP, and LO-S wrote the paper. All authors read and approved the manuscript.

## Funding

This work was supported with a grant to LO-S by the Spanish Ministry of Science and Universities of Spain (BIO2016- 77840-R) and to MP by FP7. FACCE-JPI-ERA-NET+ CLIMATE SMART AGRICULTURE (ERA46-SYBRACLYM). RS acknowledges funding from BMBF grants 031A549 (SYBRACLIM) and 0315910 (CONVIGOUR). NN, AB, LL, and GB acknowledge funding from the transnational PLANT-KBBE cooperation project CONVIGOUR with funding from ANR (France). MB was supported by a postdoctoral research fellowship and JC-C by a PhD contract (FPI), both funded by grant SEV-2016-0672 to the CBGP (Centre of Excellence Severo Ochoa Program of the Agencia Estatal de Investigación, Spain). GC-C was supported by a PhD contract by Universidad Politécnica de Madrid (UPM; programa propio).

## Conflict of Interest

The authors declare that the research was conducted in the absence of any commercial or financial relationships that could be construed as a potential conflict of interest.

## Supplementary Material

Supplementary Figure S1 Seed traits do not correlate with germination associated traits. (A) Correlation plot of seed traits showing a negative correlation of TSW, Lt0, Vt0, IS, MC and ER with seed germination traits. Data was obtained from the GEVES automated phenotyping platform. Positive and negative correlations are shown in blue and red, respectively. Size and color intensity are indicative of correlation coefficient values and when the p-value<0.05 is indicated with an asterisk (*). All data were obtained using four biological replicates (25 seeds per replicate). (B) Quantification of TSW, (C) Vt0, (D) area and (E) Lt0 from C129 (medium GR36), C033 (medium GR36) and C032 (low GR36) WOSR accessions showed significant differences in these seed parameters (C129>C032>C033) but no correlation with germination rates. Statistic analyses of the different mean values of weight, volume, area and length, were done by one-way ANOVA analysis (nonparametric). *α = 0.05; **α = 0.01; ***α = 0.001. Three replicates of 100 seeds each, from three different plants, were measured. GR (germination rate at 72 hours), GR36 (germination rate at 36 hours), MGT (mean germination time), T10 to T70 (time to reach 10% to 70% GR, respectively), FG (first germination time), ES (root elongation speed), MC (moisture content), IS (imbibition speed), VI (volume increase), CF (chlorophyll fluorescence), TSW (ten thousand seed weight), Lt0 (seed length at time 0), Vt0 (seed volume at time 0).

Supplementary Figure S2 Hormonal balance between GA, ABA and IAA regulates germination in B. napus. (A) ABA and GA metabolites are the main hormones correlating with germination rates. Pearson correlation plot of the continuous variable germination with hormone profiles of the 6 WOSR accessions. Significant correlations between germination rate (GR) and GA34, GA24, 7’OH-ABA, t-ABA, GA4, t-Z, PA, ABA and GA8 (p-val<0.05) are labelled with blue lines as well as between ABA, 7’OH-ABA, t-ABA and PA with IAA and IAA-Asp. Negative correlations between GAs and ABA are labelled with red lines. Positive and negative correlations are shown in blue and red, respectively. Size and color intensity are indicative of correlation coefficient values and when the p-value<0.05 is indicated with an asterisk (*). (B), (C) and (D) IAA metabolites differentially accumulate in WOSR seeds during germination. Stacked columns histograms showing the relative accumulation patterns of IAA (active IAA) and IAA-Asp (inactive IAA) in C129, C033 and C032 accessions, respectively. All data were obtained using three pooled biological replicates (50 seeds per replicate). For complete value data set see **Supplementary Table S1** (**datasheet 3**).

Supplementary Figure S3 Kinetics of RNA levels of genes related to ABA and GA metabolism and signaling. RNA levels according to the RNA-seq data generated in this paper for B. napus accessions C129, C33 and C32 (left to right in each graph). The five time points per accession were: dry seed, 2, 12, 36, 72 hai. Pooled samples of 30 seeds each were used with three biological replicates for each time point. B. napus gene codes and names are indicated in each graph.

Supplementary Table S1 Seed trait analysis and metabolic and hormonal profiles of WOSR accessions during germination.

Supplementary Table S2 Data of DEGs between C129, C033 and C032; k-means cluster and corresponding representative GOs.

Supplementary Table S3 List of oligonucleotides.

## References

[B1] Aguilera-Alvarado G. P. Guevara-García Á. A. Estrada-Antolín S. A. Sánchez-Nieto S. (2019). Biochemical properties and subcellular localization of six members of the HXK family in maize and its metabolic contribution to embryo germination. BMC Plant Biol. 19, 27. 10.1186/s12870-018-1605-x 30646852 PMC6332545

[B2] Albert B. Le Cahérec. F. Niogret M. F. Faes P. Avice J. C. Leport L.(2012). Nitrogen availability impacts oilseed rape (*Brassica napus* L.) plant water status and proline production efficiency under water-limited conditions. Planta 236, 659–676. 10.1007/s00425-012-1636-8 22526495 PMC3404282

[B3] Ali-Rachedi S. Bouinot D. Wagner M. H. Bonnet M. Sotta B. Grappin P. (2004). Changes in endogenous abscisic acid levels during dormancy release and maintenance of mature seeds: studies with the Cape Verde Islands ecotype, the dormant model of Arabidopsis thaliana. Planta 219, 479–488. 10.1007/s00425-004-1251-4 15060827

[B4] Bai M.-Y. Fan M. Oh E. Wang Z.-Y. (2012). A triple Helix-Loop-Helix/Basic Helix-Loop-Helix cascade controls cell elongation downstream of multiple hormonal and environmental signaling pathways in Arabidopsis. Plant Cell 24, 4917–4929. 10.1105/tpc.112.105163 23221598 PMC3556966

[B5] Barrero J. M. Piqueras P. González-Guzmán M. Serrano R. Rodríguez P. L. Ponce M. R. (2005). A mutational analysis of the ABA1 gene of Arabidopsis thaliana highlights the involvement of ABA in vegetative development. J. Exp. Bot. 56, 2071–2083. 10.1093/jxb/eri206 15983017

[B6] Barrôco R. M. Van Poucke K. Bergervoet J. H. W. De Veylder L. Groot S. P. C. Inzé D. (2013). The role of the cell cycle machinery in resumption of postembryonic development. Plant Physiol. 137, 127–140. 10.1104/pp.104.049361.1 PMC548844 15579664

[B7] Basbouss-Serhal I. Pateyron S. Cochet F. Leymarie J. Bailly C. (2017). 5′ to 3′ mRNA decay contributes to the regulation of Arabidopsis seed germination by dormancy. Plant Physiol. 173, 1709–1723. 10.1104/pp.16.01933 28126845 PMC5338662

[B8] Basnet R. K. Duwal A. Tiwari D. N. Xiao D. Monakhos S. Bucher J. (2015). Quantitative trait locus analysis of seed germination and seedling vigor in Brassica rapa reveals QTL hotspots and epistatic interactions. Front. Plant Sci. 6, 1032. 10.3389/fpls.2015.01032 26648948 PMC4664704

[B9] Bassel G. W. Mullen R. T. Bewley J. D. (2008). Procera is a putative DELLA mutant in tomato (Solanum lycopersicum): effects on the seed and vegetative plant. J. Exp. Bot. 59, 585–593. 10.1093/jxb/erm354 18250077

[B10] Becker M. G. Walker P. L. Pulgar-Vidal N. C. Belmonte M. F. (2017). SeqEnrich: a tool to predict transcription factor networks from co-expressed Arabidopsis and *Brassica napus* gene sets. PLoS One 12, e0178256. 10.1371/journal.pone.0178256 28575075 PMC5456048

[B11] Bentsink L. Koornneef M. (2008). Seed dormancy and germination. Arab. B. 6, e0119. 10.1199/tab.0119 PMC3243337 22303244

[B12] Bettey M. Finch-Savage W. E. King G. J. Lynn J. R. (2000). Quantitative genetic analysis of seed vigour and pre-emergence seedling growth traits in *Brassica oleracea*. New Phytol. 148, 277–286. 10.1046/j.1469-8137.2000.00760.x

[B13] Bewley J. D. (1997). Seed germination and dormancy. Plant Cell Online 9, 1055–1066. 10.1105/tpc.9.7.1055 PMC156979 12237375

[B14] Cao D. Cheng H. Wu W. Soo H. M. Peng J. (2006). Gibberellin mobilizes distinct DELLA-dependent transcriptomes to regulate seed germination and floral development in Arabidopsis. Plant Physiol. 142, 509–525. 10.1104/pp.106.082289 16920880 PMC1586041

[B15] Castrillo G. Turck F. Leveugle M. Lecharny A. Carbonero P. Coupland G. (2011). Speeding cis-trans regulation discovery by phylogenomic analyses coupled with screenings of an arrayed library of Arabidopsis transcription factors. PLoS One 6, e21524. 10.1371/journal.pone.0021524 21738689 PMC3124521

[B16] Cavell A. C. Lydiate D. Parkin I. Dean C. Trick M. (1998). Collinearity between a 30-centimorgan segment of Arabidopsis thaliana chromosome 4 and duplicated regions within the *Brassica napus* genome. Genome 41, 62–69. 10.1139/g97-097 9549059

[B17] Chalhoub B. Denoeud F. Liu S. Parkin I. A. Tang H. Wang X. (2014). Early allopolyploid evolution in the post-Neolithic *Brassica napus* oilseed genome. Science 345, 950–953. 10.1126/science.1253435 25146293

[B18] Chu V. T. Gottardo R. Raftery A. E. Bumgarner R. E. Yeung K. Y. (2008). MeV+R: using MeV as a graphical user interface for Bioconductor applications in microarray analysis. Genome Biol. 9, R118. 10.1186/gb-2008-9-7-r118 18652698 PMC2530872

[B19] Comai L. Dietrich R. A. Maslyar D. J. Baden C. S. Harada J. J. (2007). Coordinate expression of transcriptionally regulated isocitrate lyase and malate synthase genes in *Brassica napus* L. Plant Cell 1, 293. 10.2307/3869009 PMC159762 2535504

[B20] Davière J. M. Achard P. (2016). A pivotal role of DELLAs in regulating multiple hormone signals. Mol. Plant 9, 10–20. 10.1016/j.molp.2015.09.011 26415696

[B21] de Lucas M. Davière J. M. Rodríguez-Falcón M. Pontin M. Iglesias-Pedraz J. M. Lorrain S. (2008). A molecular framework for light and gibberellin control of cell elongation. Nature 451, 480–484. 10.1038/nature06520 18216857

[B22] Deleu C. Faes P. Niogret M. F. Bouchereau A. (2013). Effects of the inhibitor of the g-aminobutyrate-transaminase, vinyl-gaminobutyrate, on development and nitrogen metabolism in *Brassica napus* seedlings. Plant Physiol. Biochem. 64, 60–69. 10.1016/j.plaphy.2012.12.007 23370302

[B23] Demilly D. Ducournau S. Wagner M. H. Dürr C., (2014). “Digital imaging of seed germination,” in Plant Image Analysis: Fundamentals and Applications. Eds. Gupta S. D. Ibaraki Y. (Boca Ratón: CRC Press), 147–162. 10.1201/b17441-8

[B24] Derkx M. P. M. Vermeer E. Karssen C. M. (1994). Gibberellins in seeds of Arabidopsis thaliana: biological activities, identification and effects of light and chilling on endogenous levels. Plant Growth Regul. 15, 223–234. 10.1007/BF00029895

[B25] Ding L. N. Guo X. J. Li M. Fu Z. L. Yan S. Z. Zhu K. M. (2019). Improving seed germination and oil contents by regulating the GDSL transcriptional level in *Brassica napus*. Plant Cell Rep. 38, 243–253. 10.1007/s00299-018-2365-7 30535511

[B26] Dinkova T. D. Márquez-Velázquez N. A. Aguilar R. Lázaro-Mixteco P. E. (2011). Tight translational control by the initiation factors eIF4E and eIF(iso)4E is required for maize seed germination. seed Sci. Res. 21, 85–93. 10.1017/S0960258511000043

[B27] Du Z. Zhou X. Ling Y. Zhang Z. Su Z. (2010). agriGO: a GO analysis toolkit for the agricultural community. Nucleic Acids Res. 38, W64–W70. 10.1093/nar/gkq310 20435677 PMC2896167

[B28] Ducournau S. Feutry A. Plainchault P. Revollon P. Vigouroux B. Wagner M. H. (2004). An image acquisition system for automated monitoring of the germination rate of sunflower seeds. Comput. Electron. Agric. 44, 189–202. 10.1016/j.compag.2004.04.005

[B29] Ducournau S. Feutry A. Plainchault P. Revollon P. Vigouroux B. Wagner M. H. (2005). Using computer vision to monitor germination time course of sunflower (*Helianthus annuus* L.) seeds. seed Sci. Technol. 33, 329–340. 10.15258/sst.2005.33.2.06

[B30] Eastmond P. J. Graham I. A. (2001). Re-examining the role of the glyoxylate cycle in oilseeds. Trends Plant Sci. 6, 72–78. 10.1016/S1360-1385(00)01835-5 11173291

[B31] Fait A. Angelovici R. Less H. Ohad I. Urbanczyk-Wochniak E. Fernie A. R. (2006). Arabidopsis seed development and germination is associated with temporally distinct metabolic switches. Plant Physiol. 142, 839–854. 10.1002/gepi.21662 16963520 PMC1630763

[B32] Feng S. Martinez C. Gusmaroli G. Wang Y. Zhou J. Wang F. (2008). Coordinated regulation of *Arabidopsis thaliana* development by light and gibberellins. Nature 451, 475–479. 10.1038/nature06448 18216856 PMC2562044

[B33] Finch-Savage W. E. Bassel G. W. (2016). Seed vigour and crop establishment: Extending performance beyond adaptation. J. Exp. Bot. 67, 567–591. 10.1093/jxb/erv490 26585226

[B34] Finch-Savage W. E. Footitt S. (2017). Seed dormancy cycling and the regulation of dormancy mechanisms to time germination in variable field environments. J. Exp. Bot. 68, 843–856. 10.1093/jxb/erw477 28391330

[B35] Finkelstein R. R. Somerville C. R. (1990). Three classes of abscisic acid (ABA)-insensitive mutations of arabidopsis define genes that control overlapping subsets of ABA responses. Plant Physiol. 94, 1172–1179. 10.1104/pp.94.3.1172 16667813 PMC1077358

[B36] Finkelstein R. Reeves W. Ariizumi T. Steber C. (2008). Molecular aspects of seed dormancy. Annu. Rev. Plant Biol. 59, 387–415. 10.1146/annurev.arplant.59.032607.092740 18257711

[B37] Foolad M. R. Subbiah P. Zhang L. (2007). Common QTL affect the rate of tomato seed germination under different stress and nonstress conditions. Int. J. Plant Genomics 2007, 97386. 10.1155/2007/97386 18317505 PMC2246063

[B38] Galili G. (2011). The aspartate-family pathway of plants: linking production of essential amino acids with energy and stress regulation. Plant Signal. Behav. 6, 192–195. 10.4161/psb.6.2.14425 21512320 PMC3121977

[B39] Gallego-Bartolomé J. Minguet E. G. Grau-Enguix F. Abbas M. Locascio A. Thomas S. G. (2012). Molecular mechanism for the interaction between gibberellin and brassinosteroid signaling pathways in Arabidopsis. Proc. Natl. Acad. Sci. 109, 13446–13451. 10.1073/pnas.1119992109 22847438 PMC3421204

[B40] Gallego-Bartolomé J. Minguet E. G. Marín J. A. Prat S. Blázquez M. A. Alabadí D. (2010). Transcriptional diversification and functional conservation between DELLA proteins in Arabidopsis. Mol. Biol. Evol. 27, 1247–1256. 10.1093/molbev/msq012 20093430

[B41] Graeber K. Linkies A. Müller K. Wunchova A. Rott A. Leubner-Metzger G. (2010). Cross-species approaches to seed dormancy and germination: conservation and biodiversity of ABA-regulated mechanisms and the Brassicaceae DOG1 genes. Plant Mol. Biol. 73, 67–87. 10.1007/s11103-009-9583-x 20013031

[B42] Griffiths J. Murase K. Rieu I. Zentella R. Zhang Z.-L. Powers S. J. (2006). Genetic characterization and functional analysis of the GID1 gibberellin receptors in Arabidopsis. Plant Cell 18, 3399–3414. 10.1105/tpc.106.047415 17194763 PMC1785415

[B43] Gu J. Chao H. Gan L. Guo L. Zhang K. Li Y. (2016). Proteomic dissection of seed germination and seedling establishment in *Brassica napus*. Front. Plant Sci. 7, 1482. 10.3389/fpls.2016.01482 27822216 PMC5075573

[B44] Gu J. Hou D. Li Y. Chao H. Zhang K. Wang H. (2019). Integration of proteomic and genomic approaches to dissect seed germination vigor in *Brassica napus* seeds differing in oil content. BMC Plant Biol 19, 21. 10.1186/s12870-018-1624-7 30634904 PMC6329107

[B45] Han C. Yang P. (2015). Studies on the molecular mechanisms of seed germination. Proteomics 15, 1671–1679. 10.1002/pmic.201400375 25597791

[B46] Hatzig S. Breuer F. Nesi N. Ducournau S. Wagner M.-H. Leckband G. (2018). Hidden effects of seed quality breeding on germination in oilseed rape (*Brassica napus* L.). Front. Plant Sci. 9, 419. 10.3389/fpls.2018.00419 29666629 PMC5891602

[B47] Hatzig S. V. Frisch M. Breuer F. Nesi N. Ducournau S. Wagner M.-H. (2015a). Genome-wide association mapping unravels the genetic control of seed germination and vigor in *Brassica napus*. Front. Plant Sci. 6, 221. 10.3389/fpls.2015.00221 25914704 PMC4391041

[B48] Hatzig S. V. Schiessl S. Stahl A. Snowdon R. J. (2015b). Characterizing root response phenotypes by neural network analysis. J. Exp. Bot. 66, 5617–5624. 10.1093/jxb/erv235 26019255 PMC4585416

[B49] Hauvermale A. L. Tuttle K. M. Takebayashi Y. Seo M. Steber C. M. (2014). Loss of Arabidopsis thaliana seed dormancy is associated with increased accumulation of the GID1 GA hormone receptors. Plant Cell Physiol. 56, 1773–1785. 10.1093/pcp/pcv084 26136598

[B50] Hedden P. Kamiya Y. (2002). GIBBERELLIN BIOSYNTHESIS: enzymes, genes and their regulation. Annu. Rev. Plant Physiol. Plant Mol. Biol. 48, 431–460. 10.1146/annurev.arplant.48.1.431 15012270

[B51] Hedden P. Phillips A. L. (2000). Gibberellin metabolism: new insights revealed by the genes. Trends Plant Sci. 5, 523–530. 10.1016/S1360-1385(00)01790-8 11120474

[B52] Hill L. M. Morley-Smith E. R. Rawsthorne S. (2003). Metabolism of sugars in the endosperm of developing seeds of oilseed rape. Plant Physiol 131, 228–236. 10.1104/pp.010868 12529530 PMC166802

[B53] Holdsworth M. J. Bentsink L. Soppe W. J. J. (2008a). Molecular networks regulating Arabidopsis seed maturation, after-ripening, dormancy and germination. New Phytol. 179, 33–54. 10.1111/j.1469-8137.2008.02437.x 18422904

[B54] Holdsworth M. J. Finch-Savage W. E. Grappin P. Job D. (2008b). Post-genomics dissection of seed dormancy and germination. Trends Plant Sci. 13, 7–13. 10.1016/j.tplants.2007.11.002 18160329

[B55] Holman T. J. Jones P. D. Russell L. Medhurst A. Ubeda Tomas S. Talloji P. (2009). The N-end rule pathway promotes seed germination and establishment through removal of ABA sensitivity in Arabidopsis. Proc. Natl. Acad. Sci. USA 106, 4549–4554. 10.1073/pnas.0810280106 19255443 PMC2649959

[B56] Hooks M. A. Allwood J. W. Harrison J. K. D. Kopka J. Erban A. Goodacre R. (2014). Selective induction and subcellular distribution of ACONITASE 3 reveal the importance of cytosolic citrate metabolism during lipid mobilization in Arabidopsis. Biochem. J. 463, 309–317. 10.1042/bj20140430 25061985

[B57] Howell K. A. Narsai R. Carroll A. Ivanova A. Lohse M. Usadel B. (2008). Mapping metabolic and transcript temporal switches during germination in rice highlights specific transcription factors and the role of RNA instability in the germination process. Plant Physiol. 149, 961. 10.1104/pp.108.129874 19074628 PMC2633829

[B58] Hsieh W. P. Hsieh H. L. Wu S. H. (2012). Arabidopsis bZIP16 transcription factor integrates light and hormone dignaling pathways to regulate early seedling development. Plant Cell 24, 3997–4011. 10.1105/tpc.112.105478 23104829 PMC3517232

[B59] Jander G. Joshi V. (2009). Aspartate-derived amino acid biosynthesis in Arabidopsis thaliana. Arab. B. 7, e0121–e0121. 10.1199/tab.0121 PMC3243338 22303247

[B60] Kendall S. L. Hellwege A. Marriot P. Whalley C. Graham I. A. Penfield S. (2011). Induction of dormancy in Arabidopsis summer annuals requires parallel regulation of DOG1 and hormone metabolism by low temperature and CBF transcription factors. Plant Cell 23, 2568–2580. 10.1105/tpc.111.087643 21803937 PMC3226211

[B61] Kim D. H. Yamaguchi S. Lim S. Oh E. Park J. Hanada A. (2008). SOMNUS, a CCCH-type zinc finger protein in Arabidopsis, negatively regulates light-dependent seed germination downstream of PIL5. Plant Cell Online 20, 1260–1277. 10.1105/tpc.108.058859 PMC2438461 18487351

[B62] Koornneef M. Jorna M. L. Brinkhorst-van der Swan D. L. C. Karssen C. M. (1982). The isolation of abscisic acid (ABA) deficient mutants by selection of induced revertants in non-germinating gibberellin sensitive lines of Arabidopsis thaliana (L.) heynh. Theor. Appl. Genet. 61, 385–393. 10.1007/BF00272861 24270501

[B63] Kubala S. Garnczarska M. Wojtyla Ł. Clippe A. Kosmala A. Zmieńko A. (2015). Deciphering priming-induced improvement of rapeseed (*Brassica napus* L.) germination through an integrated transcriptomic and proteomic approach. Plant Sci. 231, 94–113. 10.1016/j.plantsci.2014.11.008 25575995

[B64] Kucera B. Cohn M. A. Leubner-Metzger G. (2005). Plant hormone interactions during seed dormancy release and germination. Seed Sci. Res. 15, 281–307. 10.1079/ssr2005218

[B65] Lefebvre V. North H. Frey A. Sotta B. Seo M. Okamoto M. (2006). Functional analysis of Arabidopsis NCED6 and NCED9 genes indicates that ABA synthesized in the endosperm is involved in the induction of seed dormancy. Plant J. 45, 309–319. 10.1111/j.1365-313X.2005.02622.x 16412079

[B66] Lellis A. D. Allen M. L. Aertker A. W. Tran J. K. Hillis D. M. Harbin C. R. (2010). Deletion of the eIFiso4G subunit of the Arabidopsis eIFiso4F translation initiation complex impairs health and viability. Plant Mol. Biol. 74, 249–263. 10.1007/s11103-010-9670-z 20694742 PMC2938417

[B67] Li F. Wu X. Tsang E. Cutler A. J. (2005). Transcriptional profiling of imbibed *Brassica napus* seed. Genomics 86, 718–730. 10.1016/j.ygeno.2005.07.006 16125897

[B68] Linkies A. Muller K. Morris K. Tureckova V. Wenk M. Cadman C. S. C. (2009). Ethylene interacts with abscisic acid to regulate endosperm rupture during germination: a comparative approach using Lepidium sativum and Arabidopsis thaliana. Plant Cell 21, 3803–3822. 10.1105/tpc.109.070201 20023197 PMC2814513

[B69] Liu P. P. Montgomery T. A. Fahlgren N. Kasschau K. D. Nonogaki H. Carrington J. C. (2007). Repression of AUXIN RESPONSE FACTOR10 by microRNA160 is critical for seed germination and post-germination stages. Plant J. 52, 133–146. 10.1111/j.1365-313X.2007.03218.x 17672844

[B70] Liu X. Hu P. Huang M. Tang Y. Li Y. Li L. (2016). The NF-YC-RGL2 module integrates GA and ABA signalling to regulate seed germination in Arabidopsis. Nat. Commun. 7, 12768. 10.1038/ncomms12768 27624486 PMC5027291

[B71] Liu X. Zhang H. Zhao Y. Feng Z. Li Q. Yang H.-Q. (2013). Auxin controls seed dormancy through stimulation of abscisic acid signaling by inducing ARF-mediated ABI3 activation in Arabidopsis. Proc. Natl. Acad. Sci. U. S. A. 110, 15485–15490. 10.1073/pnas.1304651110 23986496 PMC3780901

[B72] Ljung K. (2013). Auxin metabolism and homeostasis during plant development. Development 140, 943–950. 10.1242/dev.086363 23404103

[B73] Lopez-Molina L. Mongrand S. McLachlin D. T. Chait B. T. Chua N. H. (2002). ABI5 acts downstream of ABI3 to execute an ABA-dependent growth arrest during germination. Plant J. 32, 317–328. 10.1046/j.1365-313X.2002.01430.x 12410810

[B74] Lugan R. Niogret M. F. Kervazo L. Larher F. R. Kopka J. Bouchereau A. (2009). Metabolome and water status phenotyping of *Arabidopsis* under abiotic stress cues reveals new insight into ESK1 function. Plant Cell Environ. 32, 95–108. 10.1111/j.1365-3040.2008.01898.x 19054354

[B75] Lulsdorf M. M. Yuan H. Y. Slater S. M. H. Vandenberg A. Han X. Zaharia L. I. (2013). Endogenous hormone profiles during early seed development of *C. arietinum* and *C. anatolicum*. Plant Growth Regul. 71, 191–198. 10.1007/s10725-013-9819-2

[B76] Luo T. Xian M. Zhang C. Zhang C. Hu L. Xu Z. (2019). Associating transcriptional regulation for rapid germination of rapeseed (*Brassica napus* L.) under low temperature stress through weighted gene co-expression network analysis. Sci. Rep. 9, 55. 10.1038/s41598-018-37099-0 30635606 PMC6329770

[B77] Morris K. Linkies A. Muller K. Oracz K. Wang X. Lynn J. R. (2011). Regulation of seed germination in the close Arabidopsis relative *Lepidium sativum*: a global tissue-specific transcript analysis. Plant Physiol. 155, 1851–1870. 10.1104/pp.110.169706 21321254 PMC3091087

[B78] Nakabayashi K. Okamoto M. Koshiba T. Kamiya Y. Nambara E. (2005). Genome-wide profiling of stored mRNA in *Arabidopsis thaliana* seed germination: epigenetic and genetic regulation of transcription in seed. Plant J. 41, 697–709. 10.1111/j.1365-313X.2005.02337.x 15703057

[B79] Nakajima M. Shimada A. Takashi Y. Kim Y. C. Park S. H. Ueguchi-Tanaka M. (2006). Identification and characterization of Arabidopsis gibberellin receptors. Plant J. 46, 880–889. 10.1111/j.1365-313X.2006.02748.x 16709201

[B80] Nambara E. Akazawa T. McCourt P. (2008). Effects of the gibberellin biosynthetic inhibitor uniconazol on mutants of *Arabidopsis*. Plant Physiol. 97, 736–738. 10.1104/pp.97.2.736 PMC1081068 16668460

[B81] Nambara E. Marion-Poll A. (2005). Abscisic acid biosynthesis and catabolism. Annu. Rev. Plant Biol. 56, 165–185. 10.1146/annurev.arplant.56.032604.144046 15862093

[B82] Narsai R. Law S. R. Carrie C. Xu L. Whelan J. (2011). In-depth temporal transcriptome profiling reveals a crucial developmental switch with roles for RNA processing and organelle metabolism that are essential for germination in *Arabidopsis*. Plant Physiol. 157, 1342–1362. 10.1104/pp.111.183129 21908688 PMC3252162

[B83] Narsai R. Gouil Q. Secco D. Srivastava A. Karpievitch Y. V. Liew L. C. (2017). Extensive transcriptomic and epigenomic remodelling occurs during *Arabidopsis thaliana* germination. Genome Biol. 18, 172. 10.1186/s13059-017-1302-3 28911330 PMC5599894

[B84] Nguyen T. C. T. Obermeier C. Friedt W. Abrams S. R. Snowdon R. J. (2016). Disruption of germination and seedling development in *Brassica napus* by mutations causing severe seed hormonal imbalance. Front. Plant Sci. 7, 322. 10.3389/fpls.2016.00322 27014334 PMC4791391

[B85] Nishimura N. Yoshida T. Kitahata N. Asami T. Shinozaki K. Hirayama T. (2007). *ABA-Hypersensitive Germination1* encodes a protein phosphatase 2C, an essential component of abscisic acid signaling in Arabidopsis seed. Plant J. 50, 935–949. 10.1111/j.1365-313X.2007.03107.x 17461784

[B86] Nonogaki H. (2014). Seed dormancy and germinationation emerging mechanisms and new hypotheses. Front. Plant Sci. 5, 233. 10.3389/fpls.2014.00233 24904627 PMC4036127

[B87] Ogawa M. Hanada A. Yamauchi Y. Kuwahara A. Kamiya Y. Yamaguchi S. (2003). Gibberellin biosynthesis and response during Arabidopsis seed germination. Plant Cell 15, 1591–1604. 10.1105/tpc.011650.ble 12837949 PMC165403

[B88] Ogé L. Bourdais G. Bove J. Collet B. Godin B. Granier F. (2008). Protein repair L-Isoaspartyl Methyltransferase1 is involved in both seed longevity and germination vigor in *Arabidopsis*. Plant Cell 20, 3022–3037. 10.1105/tpc.108.058479 19011119 PMC2613667

[B89] Oh E. Kim J. Park E. Kim J. I. Kang C. Choi G. (2004). PIL5, a phytochrome-interacting basic helix-loop-helix protein, is a key negative regulator of seed germination in *Arabidopsis thaliana*. Plant Cell 16, 3045–3058. 10.1105/tpc.104.025163 15486102 PMC527197

[B90] Oh E. Kang H. Yamaguchi S. Park J. Lee D. Kamiya Y. (2009). Genome-wide analysis of genes targeted by PHYTOCHROME INTERACTING FACTOR 3-LIKE5 during seed germination in *Arabidopsis*. Plant Cell 21, 403–419. 10.1105/tpc.108.064691 19244139 PMC2660632

[B91] Oh E. Yamaguchi S. Kamiya Y. Bae G. Chung W. I. Choi G. (2006). Light activates the degradation of PIL5 protein to promote seed germination through gibberellin in *Arabidopsis*. Plant J. 47, 124–139. 10.1111/j.1365-313X.2006.02773.x 16740147

[B92] Oñate-Sánchez L. Vicente-Carbajosa J. (2008). DNA-free RNA isolation protocols for *Arabidopsis thaliana*, including seeds and siliques. BMC Res. Notes 1, 93. 10.1186/1756-0500-1-93 18937828 PMC2613888

[B93] Parcy F. Valon C. Raynal M. Gaubier-Comella P. Delseny M. Giraudat J. (2007). Regulation of gene expression programs during Arabidopsis seed development: roles of the *ABI3* locus and of endogenous abscisic acid. Plant Cell 6, 1567–1582. 10.2307/3869944 PMC160544 7827492

[B94] Park J. Nguyen K. T. Park E. Jeon J.-S. Choi G. (2013). DELLA proteins and their interacting RING Finger proteins repress gibberellin responses by binding to the promoters of a subset of gibberellin-responsive genes in *Arabidopsis*. Plant Cell 25, 927–943. 10.1105/tpc.112.108951 23482857 PMC3634697

[B95] Park J. Lee N. Kim W. Lim S. Choi G. (2011). ABI3 and PIL5 collaboratively activate the expression of SOMNUS by directly binding to its promoter in imbibed Arabidopsis seeds. Plant Cell 23, 1404–1415. 10.1105/tpc.110.080721 21467583 PMC3101561

[B96] Paszkiewicz G. Gualberto J. M. Benamar A. Macherel D. Logan D. C. (2017). Arabidopsis seed mitochondria are bioenergetically active immediately upon imbibition and specialize *via* biogenesis in preparation for autotrophic growth. Plant Cell 29, 109–128. 10.1105/tpc.16.00700 28062752 PMC5304351

[B97] Paterson A. H. Lan T. Amasino R. Osborn T. C. Quiros C. (2001). Brassica genomics: a complement to, and early beneficiary of, the Arabidopsis sequence. Genome Biol. 2,REVIEWS1011. 10.1186/gb-2001-2-3-reviews1011 11276431 PMC138917

[B98] Penfield S. Josse E. M. Kannangara R. Gilday A. D. Halliday K. J. Graham I. A. (2005). Cold and light control seed germination through the bHLH transcription factor SPATULA. Curr. Biol. 15, 1998–2006. 10.1016/j.cub.2005.11.010 16303558

[B99] Penfield S. MacGregor D. R. (2017). Effects of environmental variation during seed production on seed dormancy and germination. J. Exp. Bot. 68, 819–825. 10.1093/jxb/erw436 27940467

[B100] Pertea M. Kim D. Pertea G. M. Leek J. T. Salzberg S. L. (2016). Transcript-level expression analysis of RNA-seq experiments with HISAT, StringTie and Ballgown. Nat. Protoc. 11, 1650–1667. 10.1038/nprot.2016.095 27560171 PMC5032908

[B101] Piskurewicz U. Jikumaru Y. Kinoshita N. Nambara E. Kamiya Y. Lopez-Molina L. (2008). The gibberellic acid signaling repressor RGL2 inhibits Arabidopsis seed germination by stimulating abscisic acid synthesis and ABI5 activity. Plant Cell 20, 2729–2745. 10.1105/tpc.108.061515 18941053 PMC2590721

[B102] Piskurewicz U. Turečková V. Lacombe E. Lopez-Molina L. (2009). Far-red light inhibits germination through DELLA-dependent stimulation of ABA synthesis and ABI3 activity. EMBO J. 28, 2259–2271. 10.1038/emboj.2009.170 19556968 PMC2726693

[B103] Pracharoenwattana I. Cornah J. E. Smith S. M. (2005). Arabidopsis peroxisomal citrate synthase is required for fatty acid respiration and seed germination. Plant Cell 17, 2037–2048. 10.1105/tpc.105.031856 15923350 PMC1167550

[B104] Rademacher W. (1989). “Gibberellins: Metabolic pathways and inhibitors of biosynthesis,” in Target Sites of Herbicide Action. Eds. Boger Y. Sandman G. (Boca Raton: CRC Press), 128–140.

[B105] Rajjou L. Duval M. Gallardo K. Catusse J. Bally J. Job C. (2012). Seed germination and vigor. Annu. Rev. Plant Biol. 63, 507–533. 10.1146/annurev-arplant-042811-105550 22136565

[B106] Ravindran P. Verma V. Stamm P. Kumar P. P. (2017). A novel RGL2–DOF6 complex contributes to primary seed dormancy in *Arabidopsis thaliana* by regulating a GATA transcription factor. Mol. Plant 10, 1307–1320. 10.1016/j.molp.2017.09.004 28917589

[B107] Resentini F. Felipo-Benavent A. Colombo L. Blázquez M. A. Alabadí D. Masiero S. (2015). TCP14 and TCP15 mediate the promotion of seed germination by gibberellins in *Arabidopsis thaliana*. Mol. Plant 8, 482–485. 10.1016/j.molp.2014.11.018 25655823

[B108] Roberts E. H. (1973). Predicting the storage life of seed. seed Sci. Technol. 1, 499–514.

[B109] Rombolá-Caldentey B. Rueda-Romero P. Iglesias-Fernández R. Carbonero P. Oñate-Sánchez L. (2014). Arabidopsis DELLA and two HD-ZIP transcription factors regulate GA signaling in the epidermis through the L1 Box cis-element. Plant Cell 26, 2905–2919. 10.1105/tpc.114.127647 24989044 PMC4145122

[B110] Ross A. R. Ambrose S. J. Cutler A. J. Feurtado J. A. Kermode A. R. Nelson K. (2004). Determination of endogenous and supplied deuterated abscisic acid in plant tissues by high-performance liquid chromatography-electrospray ionization tandem mass spectrometry with multiple reaction monitoring. Anal. Biochem. 329, 324–333. 10.1016/j.ab.2004.02.026 15158494

[B111] Rueda-Romero P. Barrero-Sicilia C. Gómez-Cadenas A. Carbonero P. Oñate-Sánchez L. (2012). *Arabidopsis thaliana* DOF6 negatively affects germination in non-after-ripened seeds and interacts with TCP14. J. Exp. Bot. 63, 1937–1949. 10.1093/jxb/err388 22155632 PMC3295388

[B112] Sajeev N. Bai B. Bentsink L. (2019). Seeds: a unique system to study translational regulation. Trends Plant Sci. 24, 487–495. 10.1016/j.tplants.2019.03.011 31003894

[B113] Sánchez-Montesino R. Oñate-Sánchez L. (2017). “Yeast one and two hybrid high throughput screenings using arrayed libraries,” in Methods Mol. Biol, Plant gene regulatory networks. Eds. Kaufmann G. Müller-Röber B. (Dortdrecht/Heidelberg/New York: Springer), 47–65. 10.1007/978-1-4939-7125-1_5 28623579

[B114] Sánchez-Montesino R. Oñate-Sánchez L. (2018). “Screening arrayed libraries with DNA and protein baits,” in Methods Mol. Biol, Two hybrid systems: Methods and protocols. Ed. Oñate-Sánchez L. (Dortdrecht/Heidelberg/New York: Springer), 131–149. 10.1007/978-1-4939-7871-7_9 29855955

[B115] Sánchez-Montesino R. Bouza-Morcillo L. Marquez J. Ghita M. Duran-Nebreda S. Gómez L. (2019). A regulatory module controlling GA-mediated endosperm cell expansion is critical for seed germination in *Arabidopsi*s. Mol. Plant 12, 71–85. 10.1016/j.molp.2018.10.009 30419294 PMC7086157

[B116] Schwender J. Ohlrogge J. B. Shachar-Hill Y. (2003). A flux model of glycolysis and the oxidative pentosephosphate pathway in developing *Brassica napus* embryos. J. Biol. Chem. 278, 29442–29453. 10.1074/jbc.M303432200 12759349

[B117] Seo K.-I. Lee J.-H. Nezames C. D. Zhong S. Song E. Byun M.-O. (2014). ABD1 is an Arabidopsis DCAF substrate receptor for CUL4-DDB1-based E3 Ligases that acts as a negative regulator of abscisic acid signaling. Plant Cell 26, 695–711. 10.1105/tpc.113.119974 24563203 PMC3967034

[B118] Seo M. Hanada A. Kuwahara A. Endo A. Okamoto M. Yamauchi Y. (2006). Regulation of hormone metabolism in Arabidopsis seeds: Phytochrome regulation of abscisic acid metabolism and abscisic acid regulation of gibberellin metabolism. Plant J. 48, 354–366. 10.1111/j.1365-313X.2006.02881.x 17010113

[B119] Shi H. Zhong S. Mo X. Liu N. Nezames C. D. Deng X. W. (2013). HFR1 sequesters PIF1 to govern the transcriptional network underlying light-initiated seed germination in *Arabidopsis*. Plant Cell 25, 3770–3784. 10.1105/tpc.113.117424 24179122 PMC3877798

[B120] Shin Y. K. Yum H. Kim E. S. Cho H. Gothandam K. M. Hyun J. (2006). BcXTH1, a *Brassica campestris* homologue of Arabidopsis XTH9, is associated with cell expansion. Planta 224, 32–41. 10.1007/s00425-005-0189-5 16322981

[B121] Shu K. Liu X. D. Xie Q. He Z. H. (2016). Two faces of one seed: hormonal regulation of dormancy and germination. Mol. Plant 9, 34–45. 10.1016/j.molp.2015.08.010 26343970

[B122] Stavang J. A. Gallego-Bartolomé J. Gómez M. D. Yoshida S. Asami T. Olsen J. E. (2009). Hormonal regulation of temperature-induced growth in *Arabidopsis*. Plant J. 60, 589–601. 10.1111/j.1365-313X.2009.03983.x 19686536

[B123] Sun T. (2008). Gibberellin metabolism, perception and signaling pathways in *Arabidopsis*. Arab. B. 6, e0103. 10.1199/tab.0103 PMC3243332 22303234

[B124] Sun T. P. Kamiya Y. (1994). The Arabidopsis *GA1* locus encodes the cyclase ent-kaurene synthetase A of gibberellin biosynthesis. Plant Cell 6, 1509–1518. 10.1105/tpc.6.10.1509 7994182 PMC160538

[B125] Talon M. Koornneeft M. Zeevaart J. A. D. (1990). Endogenous gibberellins in *Arabidopsis thaliana* and possible steps blocked in the biosynthetic pathways of the semidwarf *ga4* and *ga5* mutants. Proc Natl Acad Sci USA. 87, 7983–7987. 10.1073/pnas.87.20.7983 2236013 PMC54876

[B126] Tam Y. Y. Epstein E. Normanly J. (2002). Characterization of auxin conjugates in *Arabidopsis*. Low steady-state levels of Indole-3-Acetyl-Aspartate, Indole-3-Acetyl-Glutamate, and Indole-3-Acetyl-Glucose. Plant Physiol. 123, 589–596. 10.1104/pp.123.2.589 PMC59026 10859188

[B127] Tatematsu K. Nakabayashi K. Kamiya Y. Nambara E. (2008). Transcription factor AtTCP14 regulates embryonic growth potential during seed germination in *Arabidopsis thaliana*. Plant J. 53, 42–52. 10.1111/j.1365-313X.2007.03308.x 17953649

[B128] Thole J. M. Beisner E. R. Liu J. Venkova S. V. Strader L. C. (2014). Abscisic acid regulates root elongation through the activities of auxin and ethylene in *Arabidopsis thaliana*. G3 4, 1259–1274. 10.1534/g3.114.011080 24836325 PMC4455775

[B129] Vázquez-Ramos J. M. de la Paz Sánchez M. (2003). The cell cycle and seed germination. seed Sci. Res. 13, 113–130. 10.1079/ssr2003130

[B130] Vishal B. Kumar P. P. (2018). Regulation of seed germination and abiotic stresses by gibberellins and abscisic acid. Front. Plant Sci. 9, 838. 10.3389/fpls.2018.00838 29973944 PMC6019495

[B131] Voegele A. Linkies A. Müller K. Leubner-Metzger G. (2011). Members of the gibberellin receptor gene family *GID1* (*GIBBERELLIN INSENSITIVE DWARF1*) play distinct roles during *Lepidium sativum* and *Arabidopsis thaliana* seed germination. J. Exp. Bot. 62, 5131–5147. 10.1093/jxb/err214 21778177 PMC3193015

[B132] Wagner M.-H. Demilly D. Ducournau S. Dürr C. Léchappé J. (2011). Computer vision for monitoring seed germination from dry state to young seedlings. seed Test. Int. 142, 49–51.

[B133] Wang Z. Chen F. Li X. Cao H. Ding M. Zhang C. (2016). Arabidopsis seed germination speed is controlled by SNL histone deacetylase-binding factor-mediated regulation of AUX1. Nat. Commun. 7, 13412. 10.1038/ncomms13412 27834370 PMC5114640

[B134] Waterworth W. M. Footitt S. Bray C. M. Finch-Savage W. E. West C. E. (2016). DNA damage checkpoint kinase ATM regulates germination and maintains genome stability in seeds. Proc. Natl. Acad. Sci. 113, 9647–9652. 10.1073/pnas.1608829113 27503884 PMC5003248

[B135] Weitbrecht K. Müller K. Leubner-Metzger G. (2011). First off the mark: Early seed germination. J. Exp. Bot. 62, 3289–3309. 10.1093/jxb/err030 21430292

[B136] Xu X. Kathare P. K. Pham V. N. Bu Q. Nguyen A. Huq E. (2017). Reciprocal proteasome-mediated degradation of PIFs and HFR1 underlies photomorphogenic development in *Arabidopsis*. Development 144, 1831–1840. 10.1242/dev.146936 28420710 PMC5450839

[B137] Yamauchi Y. Ogawa M. Kuwahara A. Hanada A. Kamiya Y. Yamaguchi S. (2004). Activation of gibberellin biosynthesis and response pathways by low temperature during imbibition of *Arabidopsis thaliana* seeds. Plant Cell 16, 367–378. 10.1105/tpc.018143 14729916 PMC341910

[B138] Ye N. Zhu G. Liu Y. Zhang A. Li Y. Liu R. (2012). Ascorbic acid and reactive oxygen species are involved in the inhibition of seed germination by abscisic acid in rice seeds. J. Exp. Bot. 63, 1809–1822. 10.1093/jxb/err336 22200664 PMC3295380

[B139] Yephremov A. Wisman E. Huijser P. Huijser C. Wellesen K. Saedler H. (1999). Characterization of the *FIDDLEHEAD* gene of *Arabidopsis* reveals a link between adhesion response and cell differentiation in the epidermis. Plant Cell 11, 2187–2201. 10.1105/tpc.11.11.2187 10559443 PMC144117

[B140] Zaharia L. I. Galka M. M. Ambrose S. J. Abrams S. R. (2005). Preparation of deuterated abscisic acid metabolites for use in mass spectrometry and feeding studies. J. Label. Compd. Radiopharm. 48, 435–445. 10.1002/jlcr.939

[B141] Zhang J. Z. Creelman R. A. Zhu J.-K. (2004). From laboratory to field. Using information from *Arabidopsis* to engineer salt, cold, and drought tolerance in crops. Plant Physiol. 135, 615–621. 10.1104/pp.104.040295 15173567 PMC514097

[B142] Zhou L. J. Mao K. Qiao Y. Jiang H. Li Y. Y. Hao Y. J. (2017). Functional identification of MdPIF1 as a Phytochrome interacting factor in Apple. Plant Physiol. Biochem. 119, 178–188. 10.1016/j.plaphy.2017.08.027 28881277

