# Supplementary figures and images for: An Integrative Approach to Analyze Seed Germination in Brassica napus
Source: Front Plant Sci. 2019 Oct 25;10:1342. doi: 10.3389/fpls.2019.01342 (PMC6824160; doi:10.3389/fpls.2019.01342)

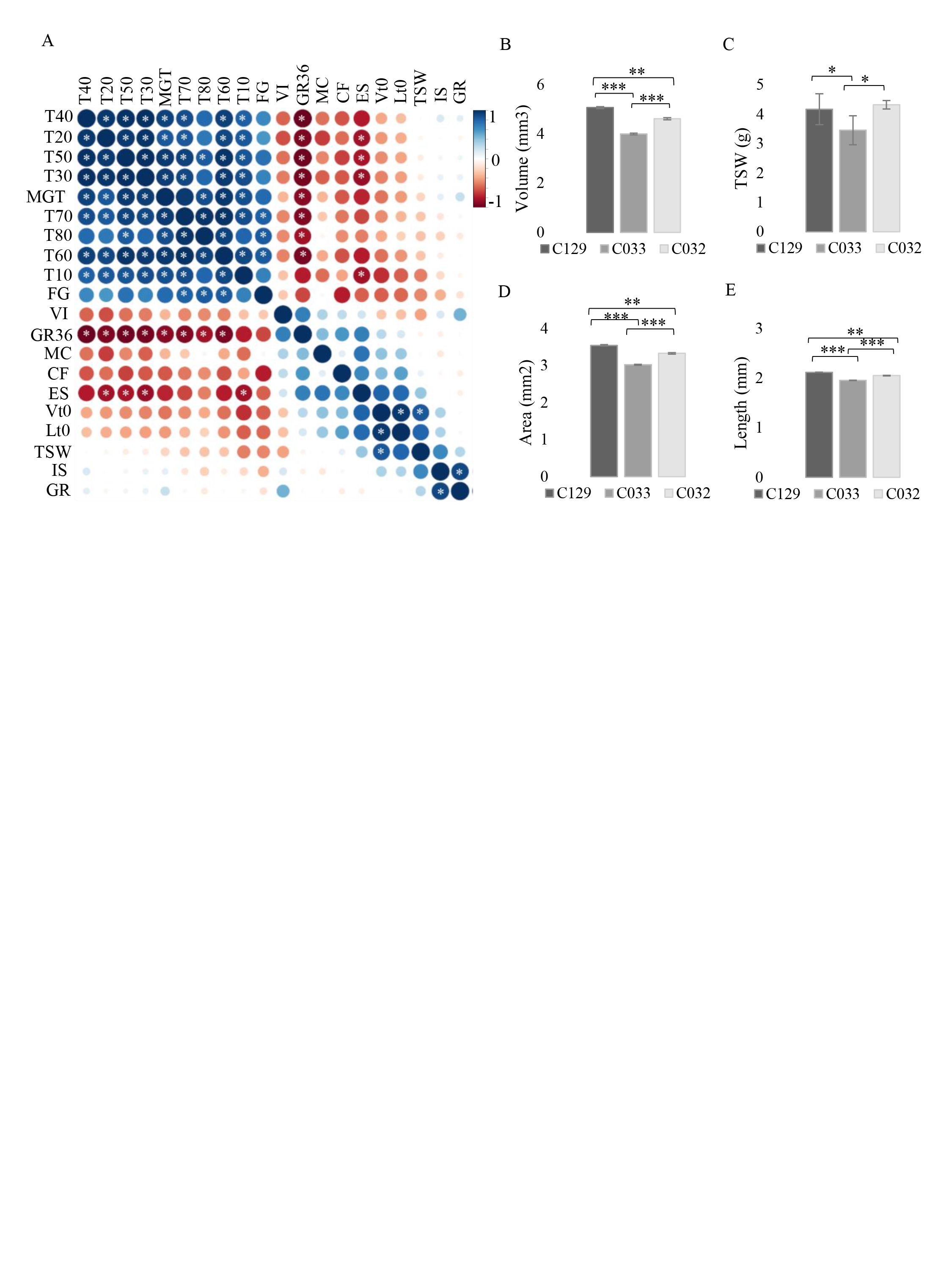

Supplement: Supplementary Figure S1 — Seed traits do not correlate with germination associated traits. (A) Correlation plot of seed traits showing a negative correlation of TSW, Lt0, Vt0, IS, MC and ER with seed germination traits. Data was obtained from the GEVES automated phenotyping platform. Positive and negative correlations are shown in blue and red, respectively. Size and color intensity are indicative of correlation coefficient values and when the p-value<0.05 is indicated with an asterisk (*). All data were obtained using four biological replicates (25 seeds per replicate). (B) Quantification of TSW, (C) Vt0, (D) area and (E) Lt0 from C129 (medium GR36), C033 (medium GR36) and C032 (low GR36) WOSR accessions showed significant differences in these seed parameters (C129>C032>C033) but no correlation with germination rates. Statistic analyses of the different mean values of weight, volume, area and length, were done by one-way ANOVA analysis (nonparametric). *α = 0.05; **α = 0.01; ***α = 0.001. Three replicates of 100 seeds each, from three different plants, were measured. GR (germination rate at 72 hours), GR36 (germination rate at 36 hours), MGT (mean germination time), T10 to T70 (time to reach 10% to 70% GR, respectively), FG (first germination time), ES (root elongation speed), MC (moisture content), IS (imbibition speed), VI (volume increase), CF (chlorophyll fluorescence), TSW (ten thousand seed weight), Lt0 (seed length at time 0), Vt0 (seed volume at time 0). [file Image_1.tif]

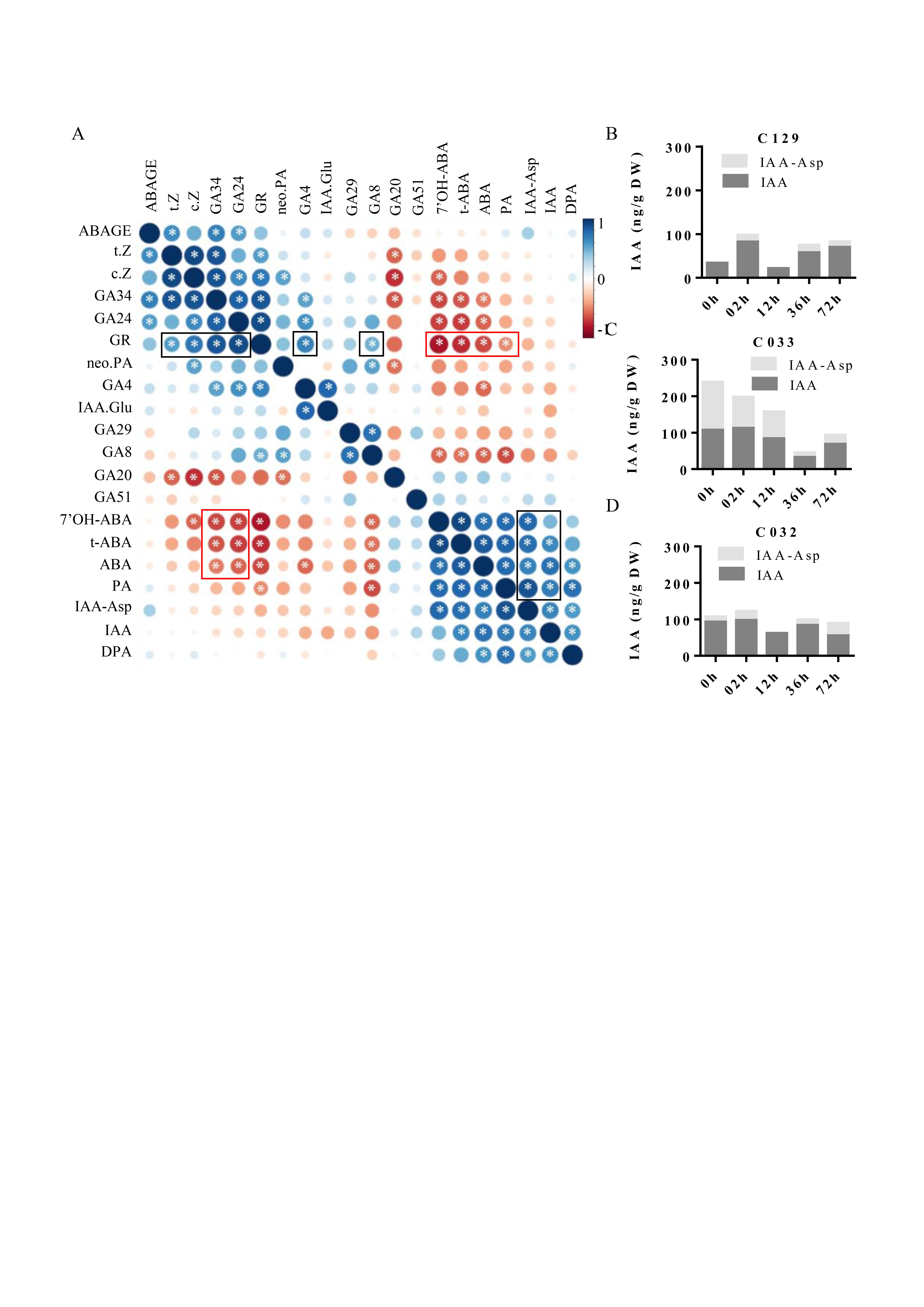

Supplement: Supplementary Figure S2 — Hormonal balance between GA, ABA and IAA regulates germination in B. napus. (A) ABA and GA metabolites are the main hormones correlating with germination rates. Pearson correlation plot of the continuous variable germination with hormone profiles of the 6 WOSR accessions. Significant correlations between germination rate (GR) and GA34, GA24, 7’OH-ABA, t-ABA, GA4, t-Z, PA, ABA and GA8 (p-val<0.05) are labelled with blue lines as well as between ABA, 7’OH-ABA, t-ABA and PA with IAA and IAA-Asp. Negative correlations between GAs and ABA are labelled with red lines. Positive and negative correlations are shown in blue and red, respectively. Size and color intensity are indicative of correlation coefficient values and when the p-value<0.05 is indicated with an asterisk (*). (B), (C) and (D) IAA metabolites differentially accumulate in WOSR seeds during germination. Stacked columns histograms showing the relative accumulation patterns of IAA (active IAA) and IAA-Asp (inactive IAA) in C129, C033 and C032 accessions, respectively. All data were obtained using three pooled biological replicates (50 seeds per replicate). For complete value data set see Supplementary Table S1 (datasheet 3). [file Image_2.tif]

Cluster 8

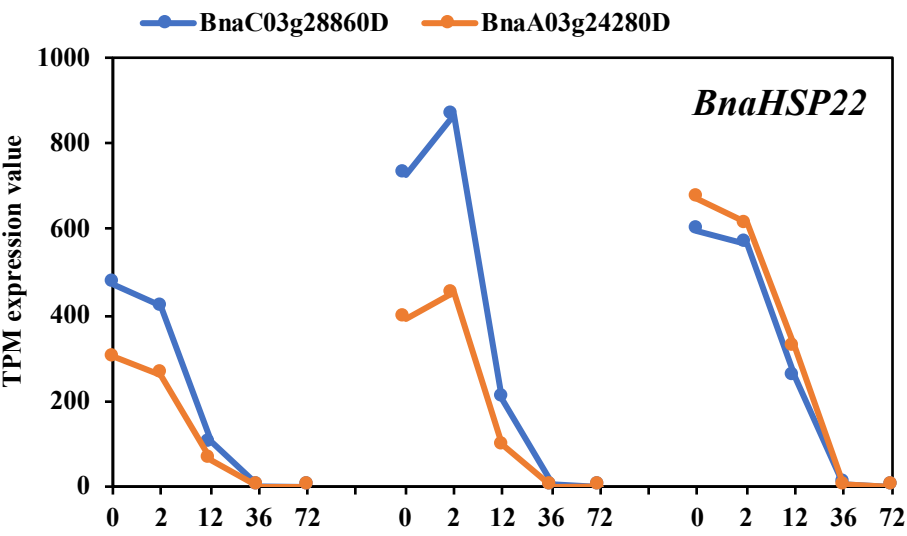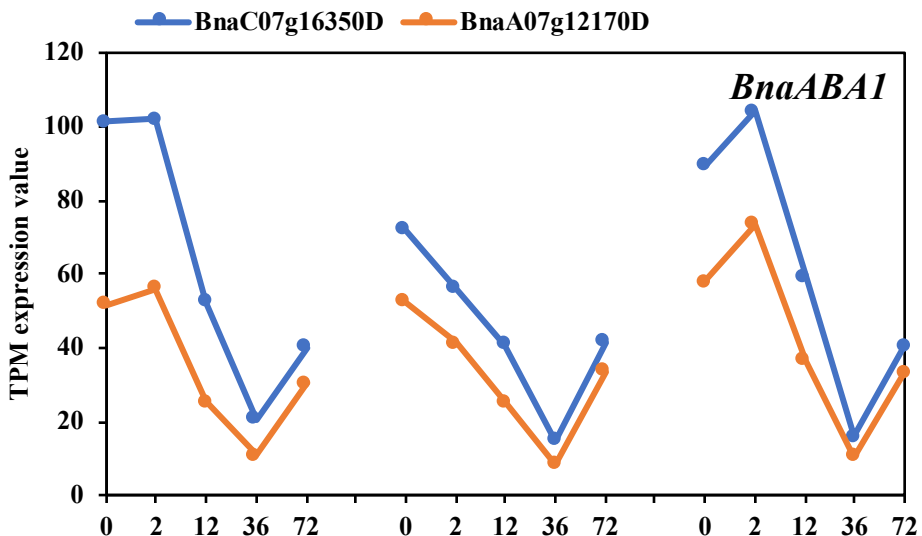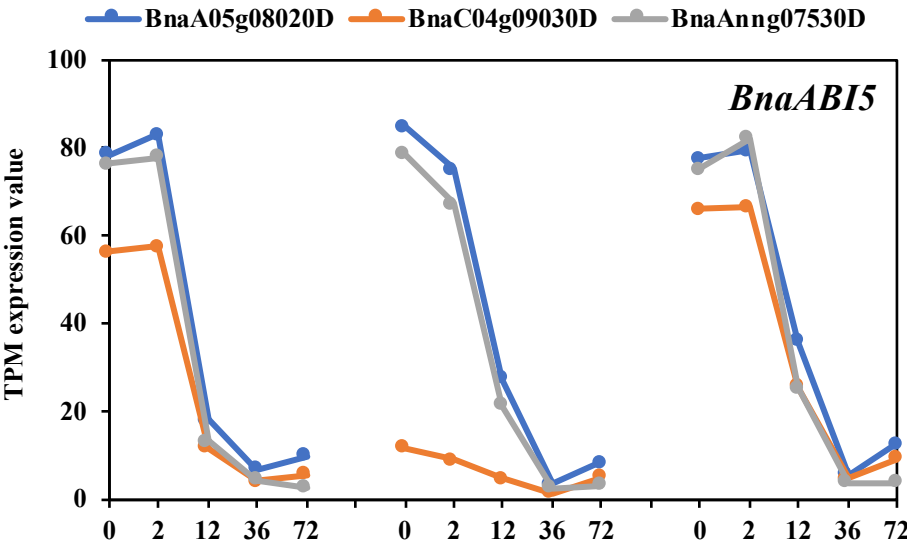

Cluster 9

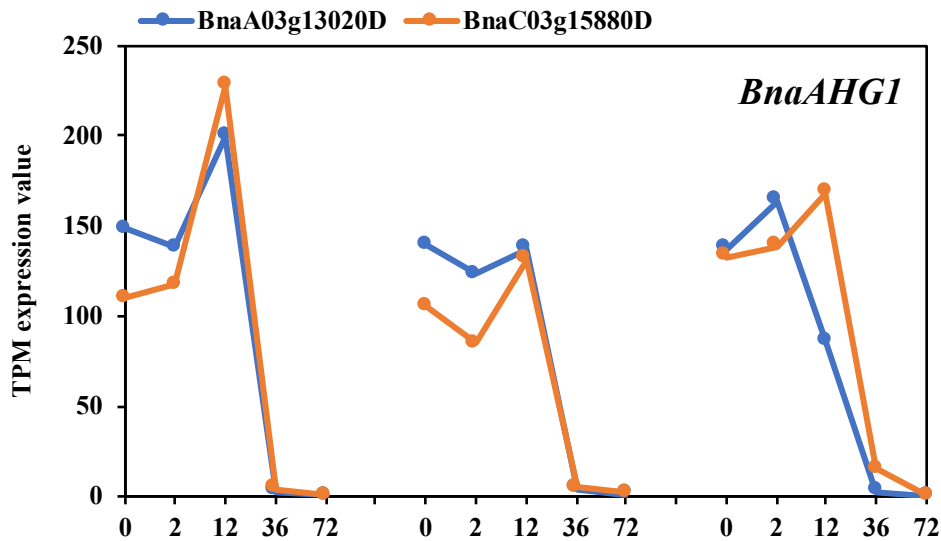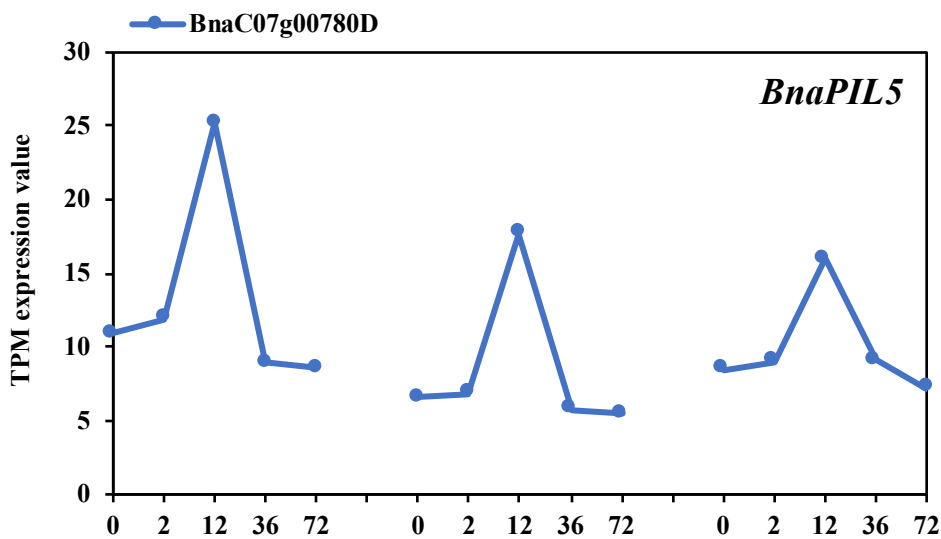

Cluster 4

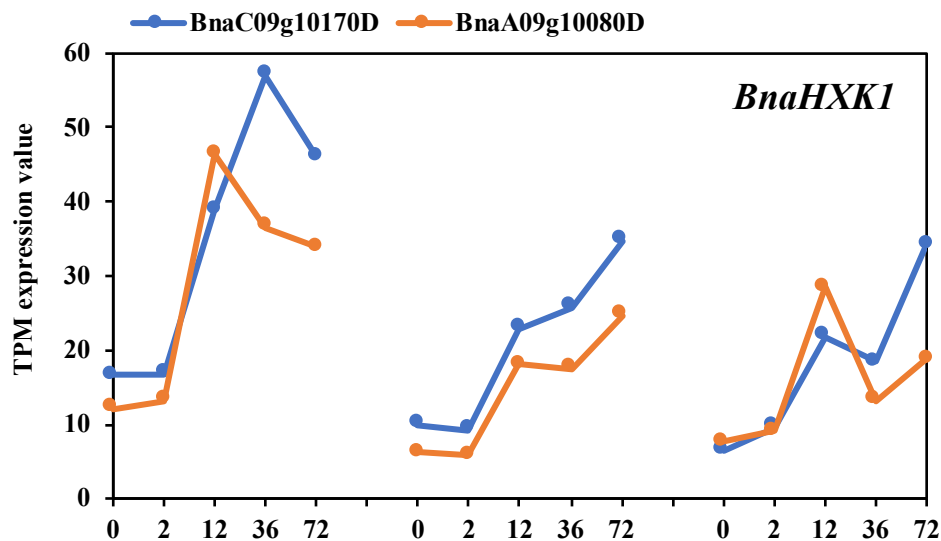

Cluster 3

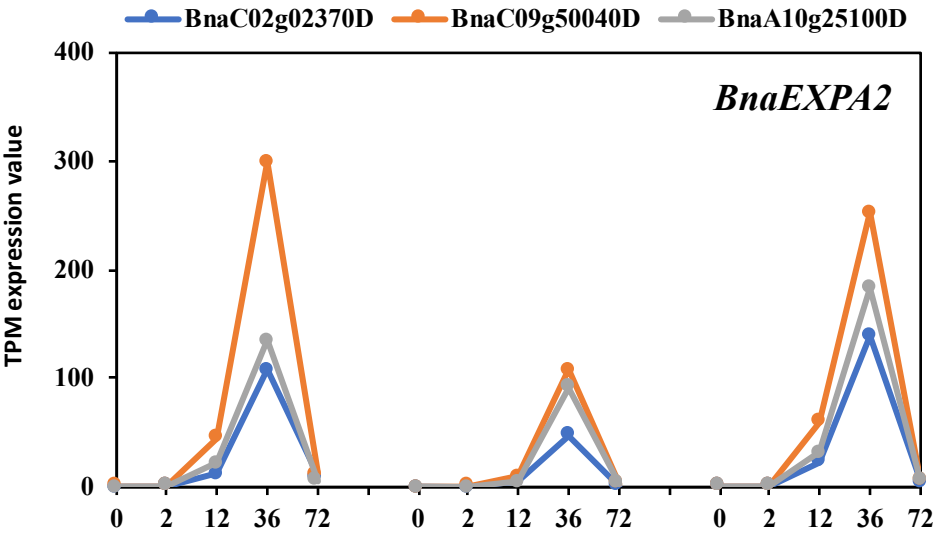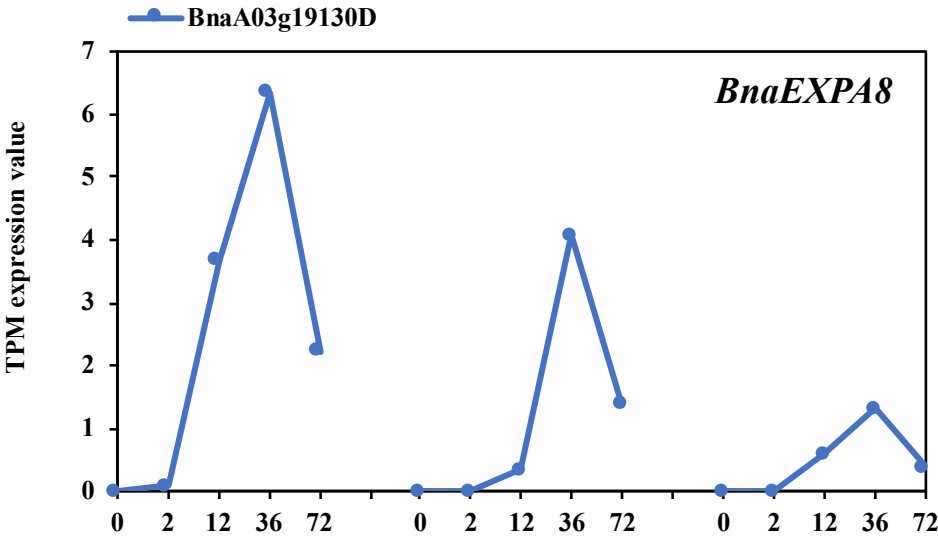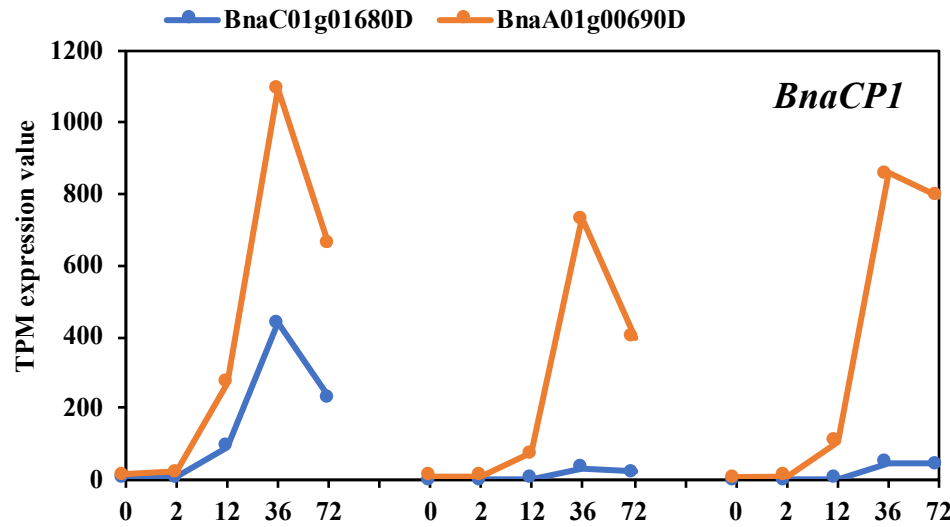

Cluster 5

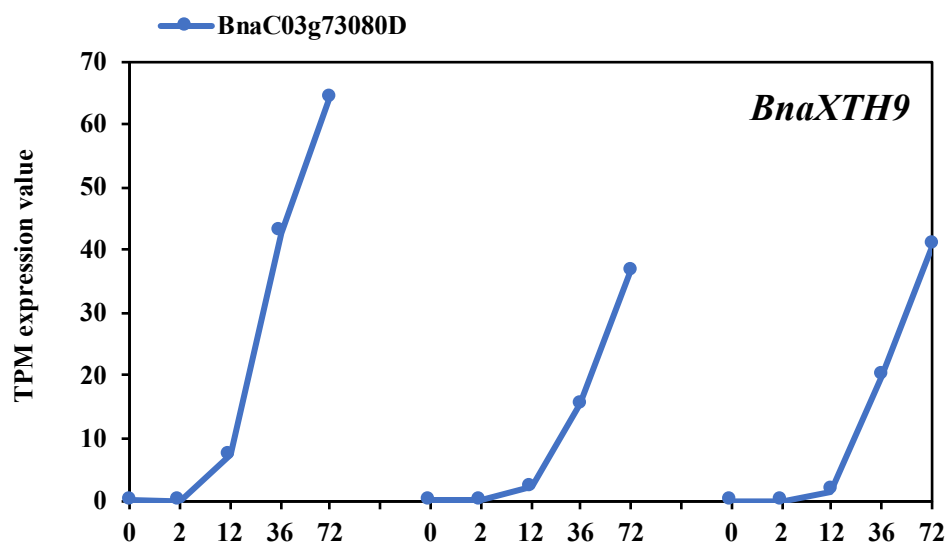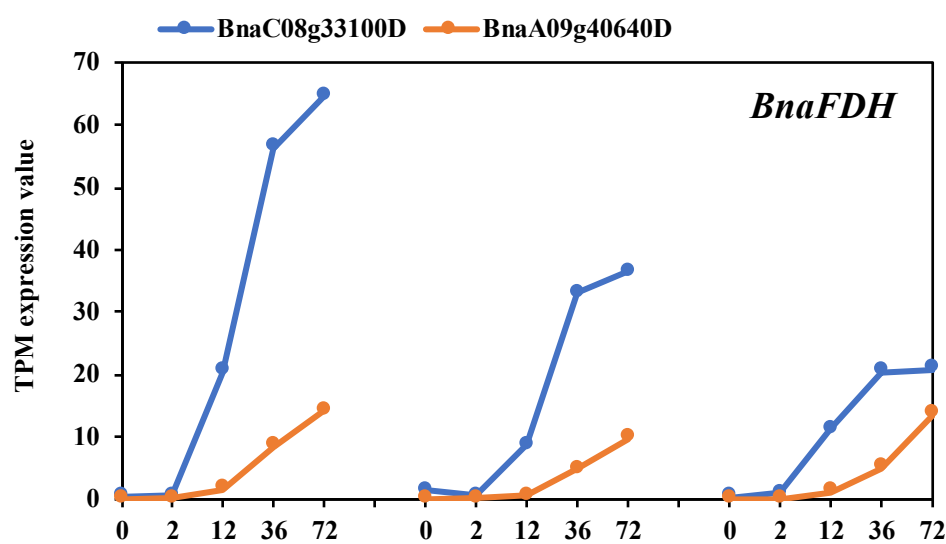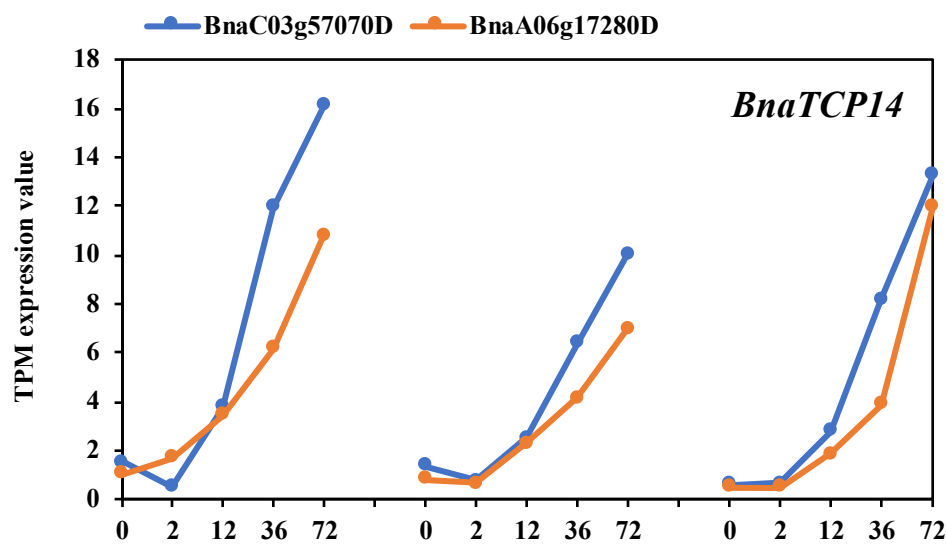

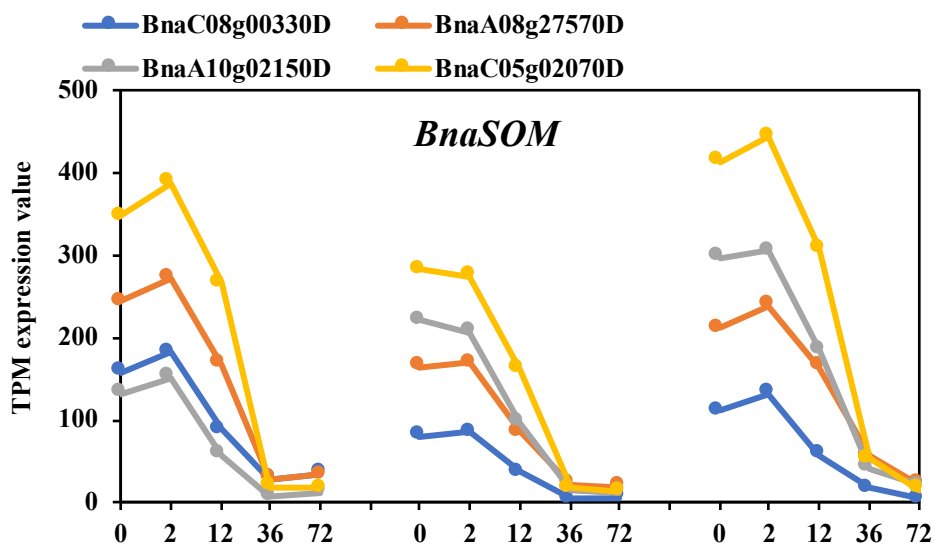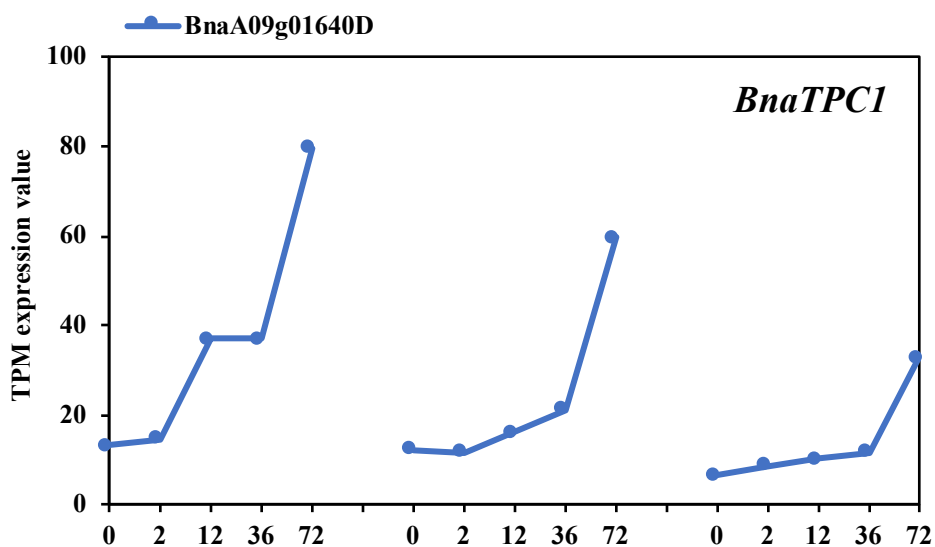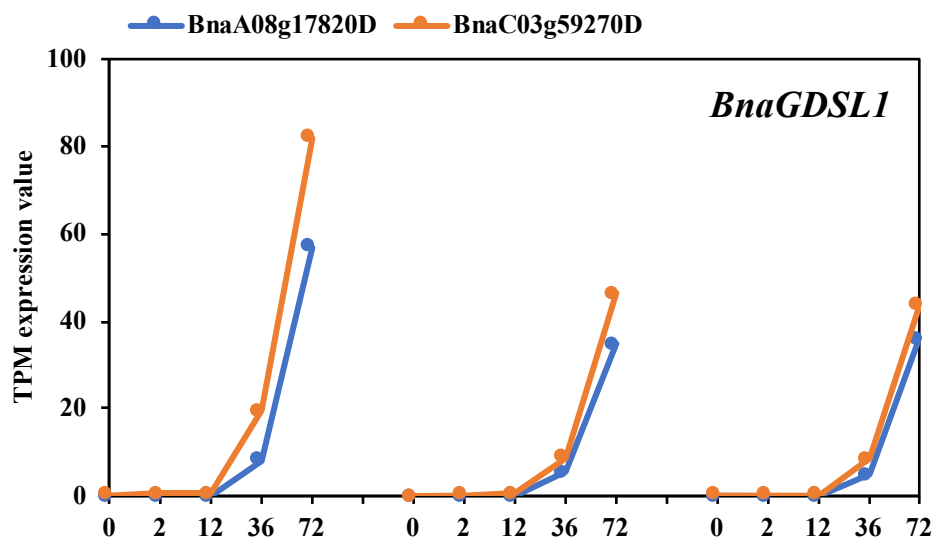

Supplement: Supplementary Figure S3 — Kinetics of RNA levels of genes related to ABA and GA metabolism and signaling. RNA levels according to the RNA-seq data generated in this paper for B. napus accessions C129, C33 and C32 (left to right in each graph). The five time points per accession were: dry seed, 2, 12, 36, 72 hai. Pooled samples of 30 seeds each were used with three biological replicates for each time point. B. napus gene codes and names are indicated in each graph. [file Image_3.pdf]
